# Longitudinal Evaluation of an N-Ethyl-N-Nitrosourea-Created Murine Model with Normal Pressure Hydrocephalus

**DOI:** 10.1371/journal.pone.0007868

**Published:** 2009-11-17

**Authors:** Ming-Jen Lee, Ching-Pang Chang, Yi-Hsin Lee, Yi-Chih Wu, Hsu-Wen Tseng, Yu-Ying Tung, Min-Tzu Wu, Yen-Hui Chen, Lu-Ting Kuo, Dennis Stephenson, Shuen-Iu Hung, Jer-Yuarn Wu, Chen Chang, Yuan-Tsong Chen, Yijuang Chern

**Affiliations:** 1 Department of Neurology, National Taiwan University Hospital, College of Medicine, National Taiwan University, Taipei, Taiwan; 2 Department of Medical Genetics, National Taiwan University Hospital, College of Medicine, National Taiwan University, Taipei, Taiwan; 3 Institute of Biomedical Sciences, Academia Sinica, Taipei, Taiwan; 4 Department of Neurosurgery, National Taiwan University Hospital, College of Medicine, National Taiwan University, Taipei, Taiwan; 5 The McLaughlin Research Institute, Great Falls, Montana, United States of America; Baylor College of Medicine, United States of America

## Abstract

**Background:**

Normal-pressure hydrocephalus (NPH) is a neurodegenerative disorder that usually occurs late in adult life. Clinically, the cardinal features include gait disturbances, urinary incontinence, and cognitive decline.

**Methodology/Principal Findings:**

Herein we report the characterization of a novel mouse model of NPH (designated p23-ST1), created by N-ethyl-N-nitrosourea (ENU)-induced mutagenesis. The ventricular size in the brain was measured by 3-dimensional micro-magnetic resonance imaging (3D-MRI) and was found to be enlarged. Intracranial pressure was measured and was found to fall within a normal range. A histological assessment and tracer flow study revealed that the cerebral spinal fluid (CSF) pathway of p23-ST1 mice was normal without obstruction. Motor functions were assessed using a rotarod apparatus and a CatWalk gait automatic analyzer. Mutant mice showed poor rotarod performance and gait disturbances. Cognitive function was evaluated using auditory fear-conditioned responses with the mutant displaying both short- and long-term memory deficits. With an increase in urination frequency and volume, the mutant showed features of incontinence. Nissl substance staining and cell-type-specific markers were used to examine the brain pathology. These studies revealed concurrent glial activation and neuronal loss in the periventricular regions of mutant animals. In particular, chronically activated microglia were found in septal areas at a relatively young age, implying that microglial activation might contribute to the pathogenesis of NPH. These defects were transmitted in an autosomal dominant mode with reduced penetrance. Using a whole-genome scan employing 287 single-nucleotide polymorphic (SNP) markers and further refinement using six additional SNP markers and four microsatellite markers, the causative mutation was mapped to a 5.3-cM region on chromosome 4.

**Conclusions/Significance:**

Our results collectively demonstrate that the p23-ST1 mouse is a novel mouse model of human NPH. Clinical observations suggest that dysfunctions and alterations in the brains of patients with NPH might occur much earlier than the appearance of clinical signs. p23-ST1 mice provide a unique opportunity to characterize molecular changes and the pathogenic mechanism of NPH.

## Introduction

Normal-pressure hydrocephalus (NPH) is an adult-onset syndrome involving non-obstructive enlargement of the cerebral ventricles [1]. Clinically, NPH is characterized by gait apraxia, urinary incontinence, and dementia [2]. Gait disturbances are the most common symptoms of NPH, and are usually the first to be observed. Postural instability and falls are frequently characteristic of the disease [3]. Parkinsonism symptoms have also been reported in approximately 11% of NPH patients [4]. A late symptom is urinary incontinence, which starts as an increase in urinary frequency and/or urgency, but progresses to incontinence [5]. Cognitive impairment tends to be predominantly subcortical in nature. Briefly, patients show various defects including slow information processing, abnormalities in memory and executive functions, reduced psychomotor activity, visuospatial deficits, and mood changes without the presence of focal cortical deficits [6], [7], [8]. Ventricular enlargement disproportionate to the cerebral atrophy without macroscopic obstruction of cerebrospinal fluid (CSF) flow is a feature of brain images. Usually, enlargement of the frontal and temporal horns is relatively uniform and symmetrical with sparing of the fourth ventricle. Shunt surgery, the most common treatment for NPH patients, is usually effective in partially ameliorating NPH symptoms, but cannot completely restore the damages caused by NPH [9]. To date, no mouse model with these clinical features of NPH has been identified.

N-Ethyl-N-nitrosourea (ENU) mutagenesis has been widely used to create a large number of germline point mutations, and is a powerful tool for creating disease models [10]. Using a combination of analyses that included magnetic resonance imaging (MRI) and rotarod performance, we screened the offspring of ENU-treated mice and identified a heritable mouse mutant (designated p23-ST1) with clinical signs similar to NPH. Further phenotypic and functional analyses suggested that p23-ST1 is a novel model for NPH with a potential causative mutation located within a 5.3-cM region on mouse chromosome.

## Results

### Brain MRI and Intracerebral Pressure of p23-ST1 Mice

**Figure 1:**
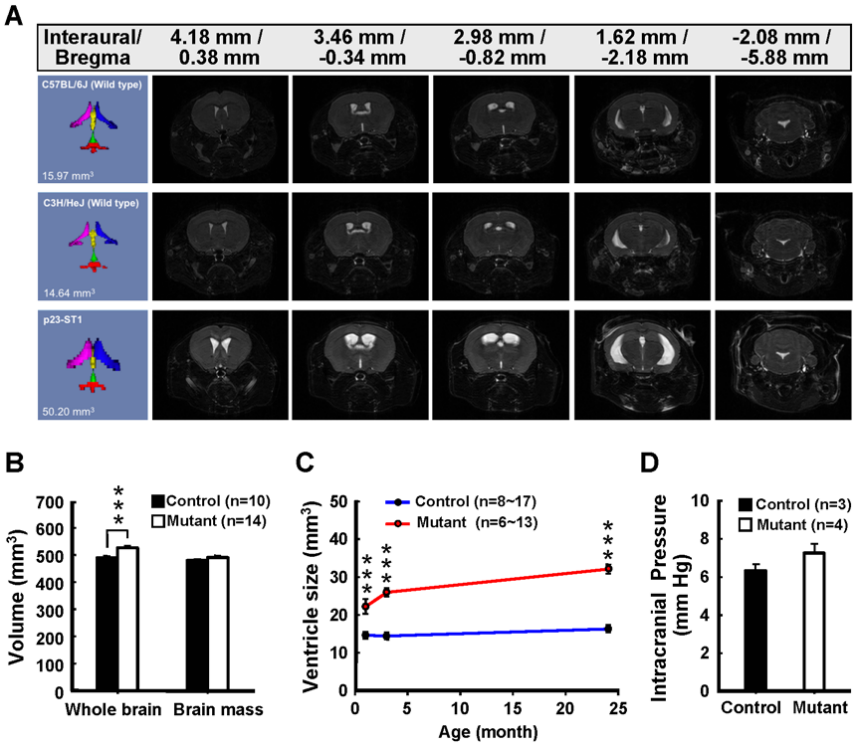
p23-ST1 mice exhibit enlarged ventricles starting from the age of 1 month. **(A)** Representative 3D brain MRI images from a wildtype C57BL/6J mouse, a wildtype C3H/HeJ mouse, and a p23-ST1 mouse at the age of 3 months. The ventricle was further delineated into the right lateral ventricle (magenta), left lateral ventricle (blue), 3^rd^ ventricle (yellow), the aqueduct (green), and 4^th^ ventricle (red). The ventricle size (mm^3^) of each brain was calculated and is shown in the left lower corner of each plate. Representative pictures of 2D MRI images (from the forebrain to the cerebellum) of each mouse are shown. **(B)** The sizes of whole brains and brain masses of mutant and control mice were assessed at the age of 3 months. The size of the brain mass was calculated by subtracting the size of the ventricle from that of the whole brain. Data were compared by Student's *t*-test (*** *p*<0.001). No significant difference in the brain masses of mutant and control mice was observed, suggesting the absence of gross brain atrophy in mutant mice. The enlarged ventricles in mutant mice apparently contributed to the slightly larger whole brains in mutant mice when compared with those in control mice. **(C)** Ventricle sizes of mutant and control mice were assessed at the ages indicated on the x-axis using 3D-MRI. The mean±SD at each point are plotted on the y-axis. Data were compared by Student's *t*-test (*** *p*<0.001). Generally, ventricular size increased with age in both mutant (red) and control (blue) individuals, but the sizes seen in the mutants statistically significantly differed from those of the controls. (D) The intracranial pressures of mutant and control mice were measured at the age of 24 months. No significant difference in the CSF pressure of mutant and control mice was found.

Micro-MRI is a non-invasive imaging tool for monitoring brain anatomy, function, and neurochemistry over time in the same animal [11]. In a screening of 128 generation-3 (G3) mice, we identified a mouse mutant (p23-ST1) with enlarged brain ventricles that proved heritable (Supplementary Fig. S1). The size of the ventricle was quantified using 3D-MRI (Fig. 1, Table 1). Mice with brain ventricles larger than the mean ventricle size plus 3 standard deviations (SDs) (Supplementary Table S1) were considered to be affected mutants. Notably, the size (16.26±2.16 mm^3^, mean±SD, *n* = 14) of the lateral ventricles (but not those of the cerebral aqueduct or 4th ventricle) in mutant mice was much larger than those of the wild-type mice (B6: 10.20±2.82 mm^3^, mean±SD, *n* = 18; C3H: 9.84±1.59 mm^3^, mean±SD, *n* = 18; control: 5.75±1.10 mm^3^, mean±SD, *n* = 11) (Fig. 1A, Table 1, Supplementary Table S1). This implies that the pathology is associated more with the cerebrum rather than the cerebellum or brainstem.

**Table 1:** 3D-MRI analysis of control and mutant mice at the age of 3 months.

	Control (mm^3^) Mean±SD; n = 11	Mutant (mm^3^) Mean±SD; n = 14
**4^th^ ventricle**	2.22±0.35	2.40±0.45
**Aqueduct**	1.50±0.41	1.44±0.30
**3^rd^ ventricle**	3.05±0.57	4.30±0.75***
**Lateral ventricle (left)**	2.87±0.68	7.78±1.28***
**Lateral ventricle (right)**	2.88±0.55	8.48±1.07***
**Total ventricle**	12.52±1.75	24.40±2.45***

Volumes of the forth ventricle, aqueduct, third ventricle, left and right lateral ventricles, and total ventricle of mutant (*n* = 14) and control (*n* = 10) mice at the age of 3 months were measured by 3D-MRI. (Mean±SD; *** *p*<0.001, Student's *t*-test).

As a consequence of ventricle enlargement, the overall size of the brains of mutant mice (525.69±24.89 mm^3^, mean±SD, *n* = 14) was larger than that of control mice (489.55±18.21 mm^3^, mean±SD, *n* = 10) (Fig. 1B). This change in ventricle size was detectable at an early age and persisted, with gradual progression, throughout life (Fig. 1C). However, these changes were not associated with a significant change in brain mass between mutant (489.83±26.97 mm^3^, mean±SD, *n* = 14) and control mice (479.10±18.06 mm^3^, mean±SD, *n* = 10) precluding the possibility of gross atrophy. No significant change in the volume of the corpus callosum between mutant (8.01±0.22 mm^3^, mean±SEM, *n* = 6) and control mice (8.10±0.45 mm^3^, mean±SEM, *n* = 6) was detected either.

The intracranial pressure (ICP) recordings in mutant mice were similar to those of control mice [7.25±0.34 mmHg (*n* = 4) and 6.33±0.23 mmHg (*n* = 3), respectively; mean±SEM, *p* = 0.21, Student's *t*-test] (Fig. 1D). This result suggests that despite an increase in ventricle volume and brain size, p23-ST1 mutant mice have NPH.

### Ventricle Enlargement and the CSF Pathway of p23-ST1 Mice

**Figure 2:**
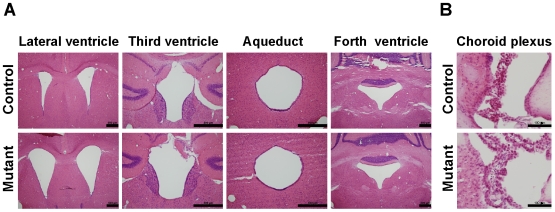
Histological assessment of the cerebrospinal fluid (CSF) pathway. (A) Serial coronal brain sections with H&E staining from 6-month-old mutant (lower panel) and age-matched control mice (upper panel) revealed enlarged lateral and third ventricles in mutant mouse compared to the control mice. The sizes of the cerebral aqueduct and fourth ventricle of the mutant mice were similar to those of control mice. Scale bars = 500 µm. **(B)** Histological analysis of the choroid plexus of mutant (lower panel) and control (upper panel) mice aged 24 months. Microscopically, no hypertrophy or tissue swelling of the choroid plexus of mutant mice was found. Representative pictures of three independent mice in each group are shown. Scale bars = 100 µm.

To determine which part of the ventricle was enlarged in p23-ST1 mutant mice, serial coronal sections along the axis of the brain were microscopically examined. Consistent with 3D-MRI analyses (Table 1), the sizes of the lateral and third ventricles of mutant mice were larger than those of control mice, while no difference in the size of the aqueduct or fourth ventricle between these two groups was observed (Fig. 2A). Collectively, no apparent obstruction in the ventricle of p23-ST1 mice was found. In addition, conventional histological analyses revealed that the choroid plexus, from which the CSF is secreted, of p23-ST1 mutant mice appeared normal when compared to that of control mice (Fig. 2B).

**Figure 3:**
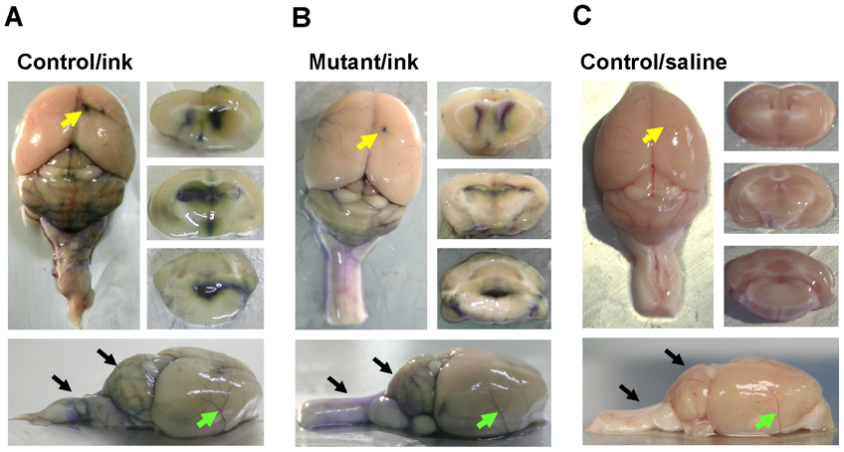
Assessment of the cerebrospinal fluid (CSF) pathway by an intraventricular injection of India ink. Mice (12 months old, *n* = 3 in each group) were intraventricularly injected with India ink. Whole-brain (upper left panel and lower panel) and serial brain sections (upper right panel) were analyzed 10 min post injection for ink distribution and diffusion in the brain of control (A) and mutant mice (B). Tissue sections from a control mouse injected with saline are shown as a negative control (C). Diffuse ink was clearly observed in the lateral, third, and fourth ventricles of both control (A) and mutant mice (B). Likewise, the darkly ink-stained vessels at the cerebrum (green arrows), cerebellum, and spinal cord (black arrows) were also evident in both mutant and control mice. No dark-blue staining was found in the brain of the negative control (C). Yellow arrows mark the injection sites. Representative pictures of three independent mice in each group are shown.

To further verify whether an obstruction existed in the CSF pathway of p23-ST1 mutant mice, a flow tracer (India ink) was intraventricularly injected into the right lateral ventricle. An obstruction in ventricles would have delayed the appearance of ink in the ventricular system as well as in ink-tinged vessels. As shown in Fig. 3, the injected ink diffused into the left lateral, third, and fourth ventricles. Furthermore, ink-tinged vessels were evident in the cerebral cortex (green arrows), cerebellum, and spinal cord (black arrows) from both control and mutant mice. No signal was found in the brain of control mice injected with saline. Taken together, these findings indicate that ventricular enlargement of p23-ST1 mice was due to a communicating hydrocephalus.

To exclude the possibility of tissue swelling in hydrocephalic mice, the brain water content was measured. The percentages of brain water were 79.3%±0.8% and 79.6%±1.2% for the control and the p23-ST1 mutant mice, respectively (mean±SEM, *n* = 4, *p* = 0.86, Student's *t-* test).

### Phenotypic Characterization of p23-ST1 Mice

Mutant mice (p23-ST1) were weighed every month starting from the age of 1 month. Despite ventricular enlargement, the general appearance and body weight (Supplementary Fig. S2) of p23-ST1 mice did not overtly differ from those of their control littermates throughout their lives. The heart rate of mutant mice was similar to those of control mice (540.0±3.5 beats/min, mean±SEM, *n* = 3; and 550.5±27.6 beats/min, mean±SEM, *n* = 3 respectively, *p = *0.72, Student's *t*-test). The mean blood pressure (BPm) of mutant mice were similar to those of control mice (83.2±3.2 mmHg; mean±SEM; *n* = 3 and 81.3±4.3mmHg; mean±SEM, *n* = 3 respectively, *p* = 0.73, Student's *t*-test). No significant differences in serum biochemistry parameters (including total cholesterol, triglyceride, glucose, magnesium, calcium, creatinine, C-reactive protein, alanine aminotransferase, creatinine kinase, amylase, and osmolarity) were found either (Supplementary Table S2).

**Figure 4:**
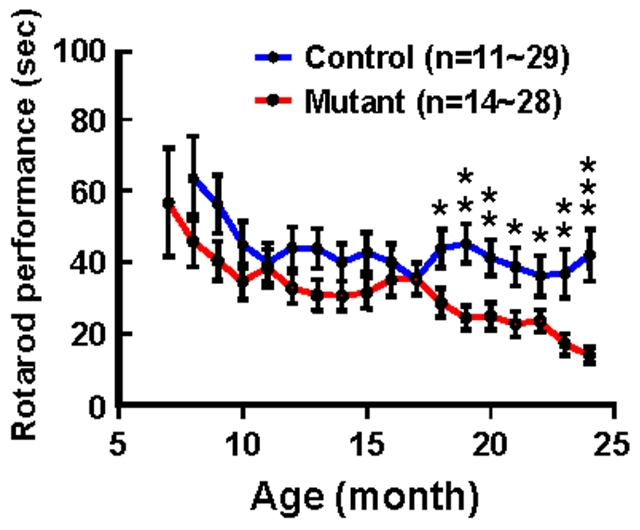
Motor coordination assessment of p23-ST1 mice by rotarod performance. Rotarod performances (at 24 rpm) of mutant (red) and control (blue) mice were evaluated monthly. These data were compared by Student's *t*-test and were found to diverge at 18 months of age (* *p*<0.05; ** *p*<0.01; *** *p*<0.001) with mutant individuals performing less well than control individuals.

**Figure 5 pone-0007868-g005:**
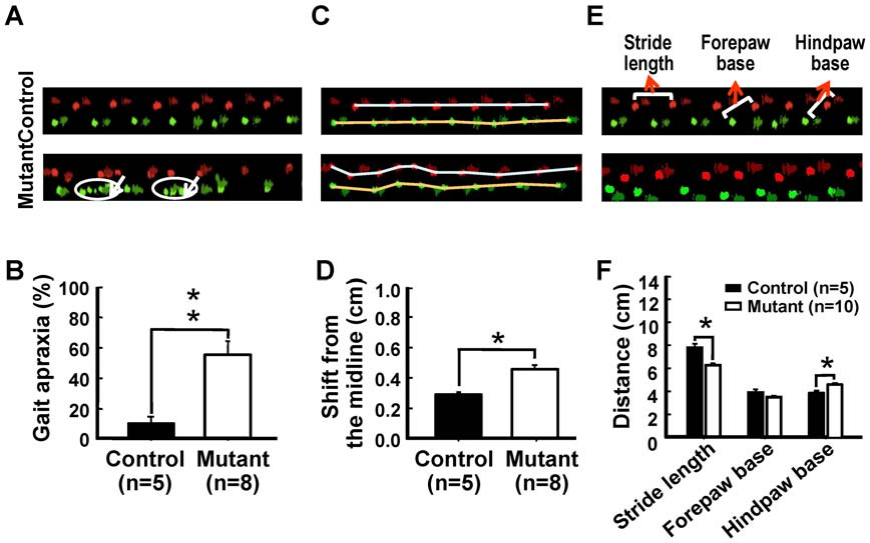
Gait analysis of p23-ST1 mice. The gait status of aged mice (20∼24 months old) was assessed by the CatWalk automatic gait analysis system. Real-time gait images of mutant and control mice are shown in panels A, C, and E. Footprint colors were assigned by the program (green, right; red, left; light, forelimbs; dark, hindlimbs). **(A)** Stumbling gait (i.e., gait apraxia, white arrows) of mutant mice (lower trace) but not control individuals (upper trace). Note that the forepaws were conspicuously affected. **(B)** Percentages of mis-placed feet in both mutant and control mice are presented graphically. The difference between the two groups was significant at *p*<0.01. **(C)** The unsteady, wobbly gait of mutant mouse compared with a control animal that moved in a straight line. The distance between the left (white line) and right (yellow line) path along with the midline was determined. **(D)** Graphical presentation of these data. The shift from the midline was more significant in mutant than control mice (*p*<0.05). To evaluate the motor performance and gait balance, the distance of the stride lengths as well as the distance between the bilateral paws were measured (**E** and **F**). Mutant mice showed a reduction in stride length and a wider base between the hind paws (*p*<0.05) **(F)**. Data are presented as the mean±SEM in each group.

From the age of 18 months, p23-ST1 mice displayed poor performance on the rotarod running test compared to control mice (Fig. 4) suggesting a decline in motor coordination. To gain insights into the nature of the motor coordination defect, mice were examined using the “CatWalk” test. This allowed us to examine the placement of their paws, the length of their stride, and the path taken along the “walk”. In this test, mutant mice displayed a more-frequent repetitive stumble (i.e., multiple attempts to place the same paw on or near the same spot) and a shortening of step length compared to the normal controls (Fig. 5A, B). By measuring the deviation in the path taken by mice from the midline of the path and the position of the feet, we were able to demonstrate that mutant mice walked with an unsteady, wobbly swaying gait compared to controls (Fig. 5C, D). Further, the stride length was smaller and base width between limb pairs was larger in mutants than those observed in controls (Fig. 5E, F). This apraxic, wide-based, small-step gait exhibited by the mutant mouse is similar to the festinating gait of NPH patients [12].

**Figure 6 pone-0007868-g006:**
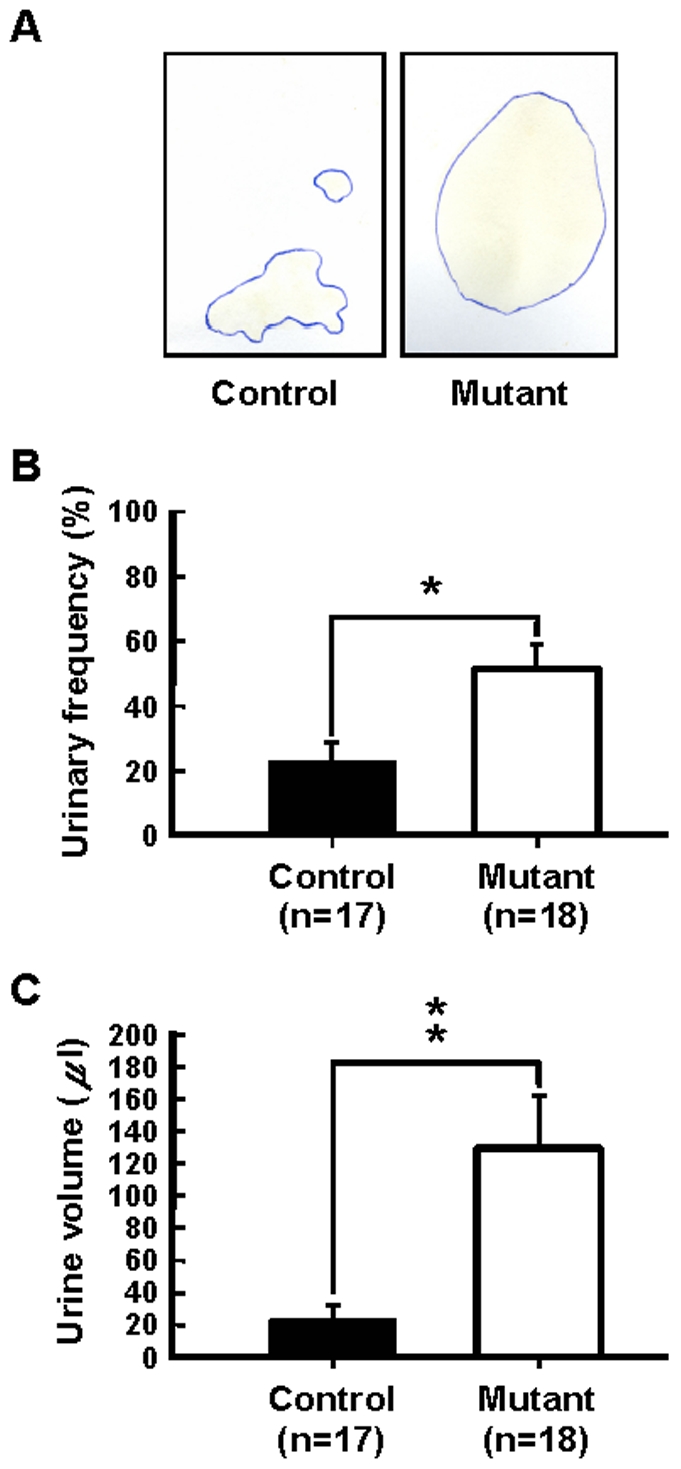
Urination dysfunction of p23-ST1 mice. (**A**) Urination of aged mice (20∼24 months old) on filter paper under mild stress (4 rpm on the rotarod) was determined. The amount of urine released was assessed by the urine-soaked areas on filter paper. Both urine frequency (**B**) and volume (**C**) were quantified. Data are presented as the mean±SEM in each group. Statistical analyses were conducted using Student's *t*-test (* *p*<0.05, ** *p*<0.01, compared to control mice).

A major feature of human NPH is urinary dysfunction or incontinence. To assess whether p23-ST1 mice might also have urinary problems in both the frequency and volume of urine produced, the urination frequency and the amount of void urine were evaluated under mild stress (detailed in Materials and Methods). Urine samples produced by the mice were collected onto chromatographic paper placed below the pivotal rod of the rotarod operated at 4 rpm for 2 min. The size of each urine spot was recorded in 12 independent tests on each mouse (Fig. 6A–C). The urinary frequencies of mutant mice were significantly greater than those of the control group (51.0%±7.4% and 22.2%±6.0%; respectively; *n* = 17 or 18, mean±SEM; *p*<0.05, Student's *t*-test). Likewise, the amount of urine (determined by measuring the size of each spot, supplementary Fig. S3) voided by the mutants was greater than that by the controls (129.1±29.9 and 23.9±10.2 µl, respectively; *n* = 17 or 18, mean±SEM, *p*<0.01, Student's *t*-test). Note that the overall urine output of an animal in each test under a minimal stress is likely to be positively correlated with the number of urination events under stress and might reflect poor sphincter control and incontinence. Collectively, these data suggest that p23-ST1 mice suffer from urinary dysfunction (incontinence) consistent with that observed in NPH patients.

**Figure 7:**
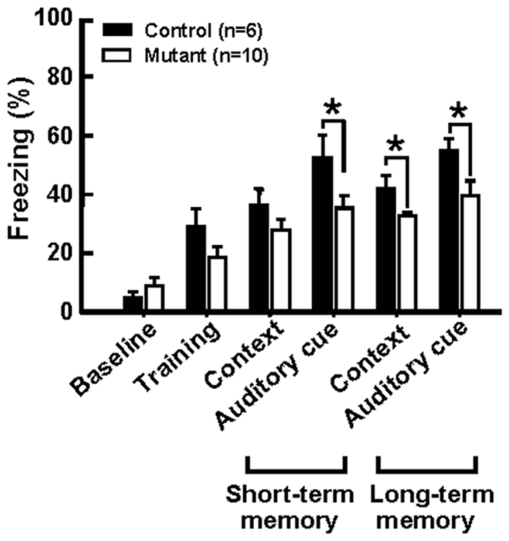
Memory impairment of p23-ST1 mice. Memory function of aged mice (18∼24 months old) was assessed by auditory fear conditioning. Both short- and long-term memory were assessed by measuring the length of time an animal froze following an auditory cue 1 (short-term) and 24 h (long-term) after initial training. Data are presented as the mean±SEM for each group. Statistical analyses were conducted using Student's *t*-test (* *p*<0.05, ** *p*<0.01, compared to control mice).

Cognitive decline and memory loss are other features associated with the human disease. We therefore attempted to determine whether mutant mice displayed a decline in mental faculties. To assess this, we employed a fear-conditioning test involving an auditory cue followed by an electric shock. After an initial training period, the mice were either returned to the test chamber for 5 min with no auditory cue (contextual test) or placed in a similar chamber with an auditory cue (memory test) 1 and 24 h after the training session. The length of time the mice spent motionless upon being placed in the chamber was recorded. These data are presented in Fig. 7. Under all tested conditions, the time taken to resume activity was shorter for mutant mice than control animals (e.g., 35.3%±4.3% and 52.6%±7.4%; respectively; *n* = 6∼10, mean±SEM; *p*<0.05, Student's *t*-test; short-term auditory-cued tests). Similar observations were also made for long-term (24 h) tests. Taken together, these data suggest that mutant mice displayed both short- and long-term memory deficits.

**Figure 8:**
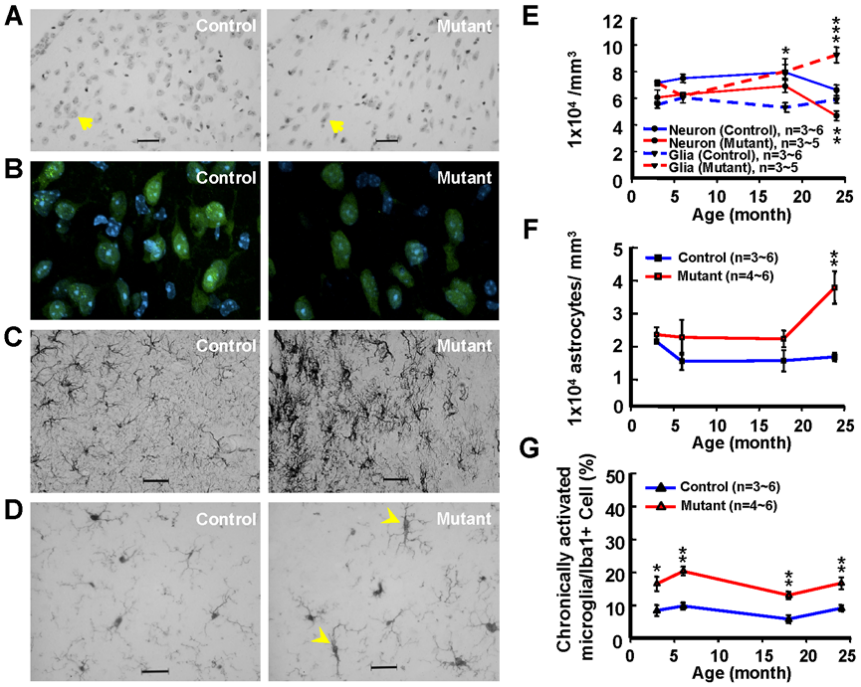
Alterations in the numbers of neurons, astrocytes, and microglia in the dorsal lateral septal nu (LSD) of p23-ST1 mice. Brain sections containing the LSD from mutant and control mice (*n* = 3∼6) were stained with Nissl stain (**A, E**), or the corresponding antibody against a neuronal marker (NeuN, **B**), an astrocyte marker (GFAP, **C**), or a microglia marker (Iba1, **D**). The density (**A**) and morphology (**B**) of LSD neurons were altered in mutant mice (**A**, arrows). Activation of astrocytes with thicker processes was also evident in mutant animals (**C, F**). The number of chronically activated microglia, characterized by their elongated and bipolar morphology with Iba1-positive immunoreactivity, was markedly increased in mutant mice (**D**; arrowheads, **G**). Representative pictures of the LSD of aged p23-ST1 mice are presented (**A, B, C, D**). A specific comparison between mutant and control mice was conducted using Student's *t*-test (* *p*<0.05, ** *p*<0.01, *** *p*<0.001). Data are presented as the mean±SEM.

Previous studies on kaolin-induced hydrocephalus in rats identified pathological changes that include neuronal cell damage and proliferation of glial cells [13], [14]. The mouse mutant was examined for pathological changes in neurons and glial cells. Nissl staining was used to establish both cell numbers and morphological changes. Among the various areas of the brain examined at 24 months of age, the septum from mutant mice displayed a marked increase in both astrocytes and microglial cells but a reduction in neuronal cells (Table 2; Fig. 8). To ascertain when these changes became manifest, we examined brain samples taken at different ages (at 3∼24 months). This study established that glial cells increased and neuronal cells decreased, within the dorsal lateral septal nucleus (LSD), at around 18 and 24 months of age, respectively (Fig. 8E). Immunohistochemical analyses identified deformed NeuN-positive neurons (Fig. 8B), increased numbers of activated astrocytes (glial fibrillary acidic protein, GFAP; Fig. 8C, F), and chronically activated microglia (ionized calcium-binding adaptor molecule 1, Iba1; Fig. 8D, G) identified by their long, rod-shaped cell bodies with thicker processes (Fig. 8D). Surprisingly, activated microglia cells were first detected in mutant mice at 3 months of age and persisted throughout life (Fig. 8G). All these changes are indicators of chronic inflammation; inflammatory responses thus occurred in p23-ST1 mice, as are found in NPH patients [15].

**Table 2:** Neuronal and glial cell numbers in the brain areas surrounding the ventricle of mutant and control mice.

	LSD		SHi		LSV		MS		Striatum		Cortex	
	Neuron	Glia	Neuron	Glia	Neuron	Glia	Neuron	Glia	Neuron	Glia	Neuron	Glia
	(N×10^4^/mm^3^)		(N×10^4^/mm^3^)		(N×10^4^/mm^3^)		(N×10^4^/mm^3^)		(N×10^4^/mm^3^)		(N×10^4^/mm^3^)	
Control (n = 6)	6.57±0.35	5.89±2.11	7.45±0.83	5.77±0.47	14.36±0.48	6.29±0.34	7.36±0.29	9.68±0.58	12.77±0.62	7.16±0.44	10.49±0.26	6.71±0.28
Mutant (n = 5)	4.61±0.36**	9.12±0.63***	5.43±0.59	6.42±0.82	12.31±1.19	8.21±0.71*	5.63±0.52*	9.87±0.68	9.52±0.52**	10.75±0.21***	9.07±0.51*	7.62±0.15*

To visualize neurons and glial cells, brain sections from mutant and control mice (24 months old, *n* = 5 or 6) were stained with Nissl stain. Examples of the results obtained are shown in Fig. 8. Student's *t*-test was used to compare the data (mean number of cells±SEM) from mutant and control individuals (* *p*<0.05; ** *p*<0.01;*** *p*<0.001). LSD, lateral septal nucleus, dorsal; SHi, septohippocampal nucleus; LSV, lateral septal nucleus, ventral; and MS, medial septal nucleus.

### Genetic Characterization of Affected p23-ST1 Mice

To map the causative mutation, p23-ST1 mice (B6 genetic background) were outcrossed with C3H mice, because of their genealogical distance and similarities in ventricle size (Supplementary Table S1). To determine the inheritance pattern, four mutant mice (either homozygous or heterozygous for the mutation) were out-crossed to wild-type C3H mice. A significant portion (35 of 45, 77.8%) of the offspring exhibited enlarged ventricles (ventricle size ≥ mean +3 SDs). This percentage is much higher than what would be expected if the mutation arose *de novo*, or was inherited as either a recessive or sex-linked gene. Thus, the mode of inheritance is consistent with an autosomal dominant trait displaying variable penetrance.

**Figure 9:**
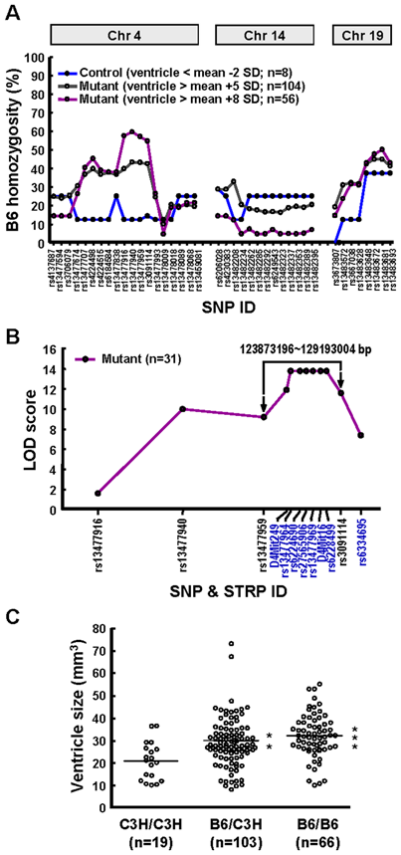
A region on chromosome 4 carries the susceptibility gene for hydrocephalus. p23-ST1 mice with a B6 genetic background were outcrossed to C3H/HeJ mice and genotyped for the presence of B6 alleles across the genome (Supplementary Fig. S4). Chromosomes with the greatest proportion of associated B6 homozygosity were identified **(A)**. By far the greatest proportion of B6 homozygosity (60%) in individuals with extreme phenotypes (≥ mean +8 SDs, *n* = 56; purple), was associated with a region on chromosome 4 between rs13477916 and rs3091114. Control individuals (i.e., those with a ventricular volume of <2 SDs from the mean) had only around 12% B6 homozygosity for the same region (blue), whereas the moderate phenotype (ventricle size ≥ mean +5 SDs) had about 40% (gray). The other two likely chromosomal regions on 14 and 19, identified in the initial genome scan (Supplementary Fig. S4), yielded much-smaller differentials among the three groups. **(B)** LOD scores at the linked locus on chromosome 4 from mutant mice. Under the assumption of autosomal dominant transmission and using an intercrossing scheme, LOD scores at the linked locus on chromosome 4 were calculated using the MapManager QTX software in 31 mutant mice with extremely large ventricles (≥ mean +8 SDs). **(C)** Ventricular volumes in mice that were C3H or B6 homozygotes or B6/C3H heterozygotes in the region defined by rs13477959 and rs3091114 on chromosome 4. The mean ventricle sizes of the homozygous B6/B6 and heterozygous B6/C3H mice were significantly larger compared to mice with the homozygous C3H/C3H genotype (** *p*<0.01, *** *p*<0.001, Student's *t*-test).

To map the disease gene, B6/C3H F1 mutant mice were inter-crossed to produce F2 offspring. Mutant F2 offspring were out-crossed to C3H, and the mutant offspring from this cross were backcrossed to C3H. Mutant backcrossed offspring were then inter-crossed to produce mutant F2 offspring that were again backcrossed to C3H. This breeding program was repeated three more times, and the offspring were used in the mapping studies. Given this breeding strategy and assuming an autosomal dominant mode of inheritance, the percentage of B6 alleles at linked loci in mutant mice should have been greater than those present in control mice. For the initial mapping study, a subset 18 mutant mice (with ventricles ≥ mean +8 SDs), and 11 control mice (with ventricles ≤ mean −2 SDs) were subjected to a genome-wide SNP screening comprising 287 markers. From this analysis, the greatest frequency of B6 alleles was observed on chromosomes 4, 7, 14, and 19 in mutant mice (Supplementary Fig. S4). The involvement of chromosome 7 was excluded because the B6 genotype frequency of control mice was also as high as mutant mice at 90% (Supplementary Fig. S4). Further allelic frequency analyses with an expanded population (*n* = 64) showed that the association was more prominent for chromosome 4 than chromosome 14 or 19 (Supplementary Fig. S5). A higher difference in the percentage of homozygosity (B6/B6) between the moderate phenotype (ventricle size ≥ mean +5 SDs) and the extreme phenotype (≥ mean +8 SDs) was also noted for chromosome 4 (Fig. 9A). For dominant mutations, gene dosage is a common factor affecting the phenotype, in that homozygotes, if viable, are more likely to exhibit extreme characteristics. To test this hypothesis, we compared the phenotype with the genotype and observed that affected individuals with ventricle sizes of greater than the mean +5 SDs were more often heterozygous for alleles in the disease gene region, whereas those with ventricles larger than the mean +8 SDs were more often homozygous (homozygosity of 41% and 56% respectively, Fig. 9A). In contrast, control mice only exhibited 13% homozygosity for B6 alleles (Fig. 9A). This phenomenon was not observed for other chromosomes implicated in the initial linkage studies (i.e., chromosomes 14 and 19). The inclusion of additional micro-satellite markers in the region defined by rs13477916 and rs3091114 further refined the interval to suggest that the disease locus lay in a 5.3-cM region between rs13477959 and rs3091114 (Supplementary Fig. S6, Table S2). With an LOD score of 14 (Fig. 9B), the association of the disease gene with this region of chromosome 4 was highly significant.

Given the complexity of the linkage analysis, reverse mapping was used as a reciprocal approach to confirm the linkage. We compared ventricle sizes among the three groups of mice: homozygous for B6/B6, heterozygous for B6/C3H, and homozygous for C3H/C3H in the candidate region (rs13477959∼rs3091114). As shown in Fig. 9C, ventricle sizes of mice harboring at least one B6 allele were significantly larger than those that were homozygous for C3H alleles. In addition, 142 of 151 (94%) mutant mice shown in Fig. 9C had at least one B6 allele in the candidate region, further supporting our hypothesis that the responsible mutant gene is linked to this 5.3-cM region on mouse chromosome 4. The other 6% of mutant mice which had enlarged ventricles but were homozygous for the C3H loci might have resulted from spontaneous mutations [16] or natural variations in ventricle size.

## Discussion

In the present study, we identified an ENU-induced mutant mouse with hereditary ventriculomegaly. The cardinal clinical features were gait apraxia and ataxia, urinary incontinence, and cognitive decline (Figs. 4–7). Although p23-ST1 mice exhibit enlarged ventricles, ICP monitoring showed that they have normal ICP. The phenotype was transmitted in an autosomal dominant type with partial penetrance. Our linkage data suggest that the disease locus maps to a 5.3-cM region of mouse chromosome 4 between rs13477959 and rs3091114. Although the mode of transmission might not follow a simple dominant Mendelian trait, using a model-free association study, a 2 by 2 comparison of the data still provided evidence of genetic linkage to the 5.3-cM region on chromosome 4 (*χ^2^ = *16.14, *p*<0.001, Supplementary Table S3). The disease odds ratio and relative risk ratio were 2.96 and 1.52, respectively, further strengthening its association with hydrocephalus.

A few autosomal recessive mutations leading to hydrocephalus were previously reported in mice [17], [18]. Furthermore, mutations in two different genes (*Foxc1* and *Lmx1a*) were found to be responsible for two hydrocephalus mouse models, congenital hydrocephalus (*ch*) and dreher (*dr*) mice, respectively [19], [20]. Nonetheless, in addition to congenital hydrocephalus, defects in other organs (such as the cardiovascular system, bones, and kidneys) were also found in these congenital mutant or genetically modified mouse models [21], [22], [23], which rather differs from p23-ST1 mice or NPH patients. In addition, none of the genetic loci for these models are located on chromosome 4. The spontaneously mutated hydrocephalus-3 (hy3) mouse is another interesting hydrocephalus mouse model which exhibits hydrocephalus, nasal discharge, and runting [24], [25]. The causative mutation is currently unknown, but was mapped to mouse chromosome 8 [26]. In addition to mouse models, a rat hydrocephalus model (the H-Tx rat) with an onset during late gestation was reported [27]. Interestingly, the inheritance mode of the H-Tx rat is rather complex. Quantitative trait locus mapping revealed that the hydrocephalus trait loci of the H-Tx rat were located on four different chromosomes (9, 10, 11, and 17), which differ from the linked mutant region (orthologous to the rat chromosome 5q36) in p23-ST1 mice. Taken together, p23-ST1 is a new rodent model of hydrocephalus.

Pathological characterization of p23-ST1 mice showed marked astroglial and microglial reaction as well as a significant loss of neurons in septal areas (Fig. 8). Several neuropathological findings associated with human and experimental hydrocephalus revealed focal destruction of the ependyma, changes in the cerebral vasculature, and ensuing damage to axons and oligodendrocytes around the ventricles [13], [28]. Reactive changes in glial populations were also reported in subependymal zones [13], [28]. These pathological alterations might result from a combination of mechanical distortion and impaired cerebral blood flow. Interestingly, the gliosis associated with degenerative hydrocephalus can be ameliorated by shunt treatment [29]. In the hydrocephalic brain (post-hemorrhagic and congenital types), an astroglial reaction is a frequently encountered pathology within the periventricular white matter which is similar to what we identified in the mutant mice [30]. In addition to an increase in astrocytes, the number of chronic reactive microglia cells was also markedly elevated in the periventricular region of affected mice (Fig. 8D, G). Similar findings were reported in congenital hydrocephalus of both humans and rats [31], [32]. Specifically, Ulfig et al. [32] reported numerous macrophages located focally along the ependymal lining of the lateral ventricle, and intense microglial activity within the periventricular regions of the parenchyma in fetal hydrocephalus brains. Increasing evidence indicates that the appearance of chronically activated microglia reflects a sustained local inflammatory response. Since chronically activated microglia were found in the septal area of p23-ST1 mice at a relatively young age (3 months old) and throughout their lives, this phenomenon seems to play a critical role in NPH. Our finding that microglial activation occurred in brains of p23-ST1 mice is consistent with an earlier study describing how the levels of an inflammatory marker (tumor necrosis factor-α) in the CSF of NPH patients are higher than those in the normal population [15]. Early pathological changes preceding a long latency period prior to the emergence of clinical symptoms is not an uncommon feature in neurological diseases. Huntington's disease provides an example of early pathological changes affecting the caudate nuclei which predate the occurrence of clinical signs [33]. Therefore, we anticipate that further investigations on the inflammatory responses in the periventricular region of p23-ST1 mice will greatly increase our understanding of the NPH pathogenesis.

To date, patients with NPH can only be diagnosed after major clinical signs have appeared. Due to a lack of appropriate animal models, the timing of onset of ventricle enlargement and neurochemical abnormalities in NPH has never been determined. In 1964, McHugh described the autopsies of several patients with congenital hydrocephalus who had become symptomatic at an older age [34]. Graff-Radford and Godersky later found that three of 30 NPH patients had a head size at or above the 98^th^ percentile [35], thus concluding that NPH occurred as a result of decompensation of arrested congenital hydrocephalus. In a more-recent report, Krefft et al. [36] showed that the head sizes of both male and female patients with NPH are statistically larger than those of the normal controls. Collectively, a significant proportion of patients with NPH may have congenital hydrocephalus that becomes symptomatic later in life which is reminiscent of McHugh's conclusion. It is very likely that dysfunctions and alterations in the brains of patients with NPH might occur long before the appearance of clinical signs. p23-ST1 mice therefore provide an unique opportunity for characterizing the molecular changes and the pathogenic mechanism of NPH.

Familial cases of congenital hydrocephalus have occasionally been reported in humans and may result from defects in single genes or multifactorial determinants [37]. The most common form of hydrocephalus is X-linked hydrocephalus which is usually associated with stenosis of the aqueduct of Sylvius, and the gene responsible for this disease (L1CAM) was identified [38]. Nevertheless, other forms of congenital hydrocephalus are mostly multifactorial with a recurrence risk of about 4% [39]. Familial adult-onset cases are unusual, and the genetic basis remains obscure. To date, only two cases of familial hydrocephalus have been reported with an autosomal dominant mode of inheritance. Varadi et al. [39] described two siblings with apparent hydrocephalus that exhibited late-onset gait disturbance, urinary frequency, and cognitive impairment. Both patients markedly improved after the shunt procedure. Chalmers et al. [40] recently reported a family in which the presumed mode of inheritance was autosomal dominant with variable penetrance. These observations demonstrate that there are human forms of NPH that exhibit an autosomal dominant mode of inheritance with variable penetrance. Our mouse model of this disease syndrome recapitulates the clinical signs in these individuals and is likely to prove extremely useful in defining the disease pathology of this human syndrome. Our genetic study should eventually lead to the identification of the disease gene which could be used to screen human patients with NPH.

## Materials and Methods

### Ethics Statement

All animal experiments performed were reviewed and approved by the Institutional Animal Care and Use Committee of Academia Sinica.

### Generation of ENU-Mutated Mice

Offspring from ENU-mutated C57BL/6J (B6) mice were generated as previously detailed [41]. In brief, B6 male mice were injected intraperitoneally with three consecutive weekly injections of ENU (100 mg/kg). Following their recovery to fertility, they were crossed to B6 females to produce generation one (G1) males. The G1 males were further crossed to B6 females to produce G2 females. These G2 females were backcrossed to their sires. The offspring (G3) from these backcrosses were subjected to a phenotype screening. All of these mice were generated in the Mouse Mutagenesis Core Program Facility and were maintained in the animal core facility of the Institute of Biomedical Sciences, Academia Sinica, Taipei, Taiwan. Food and water were available *ad libitum*. C3H/HeJ (C3H) mice were purchased from the National Laboratory Animal Center, Taipei, Taiwan.

### Intracerebral Pressure (ICP) Monitoring

Mice (24 months old) were anesthetized by an intraperitoneal injection of sodium pentobarbital (80 mg/kg). Sodium pentobarbital was chosen because it is an anesthetic commonly used in animal studies for measuring intracranial pressure [42], [43]. ICP was measured by drilling a small hole (3 mm in diameter) through the skull and inserting a CODMAN MICROSENSOR ICP transducer connected to an ICP EXPRESS monitor (Johnson and Johnson Medical, Raynham, MA, USA) into the frontal lobe of the brain. After a recording period of 5 min, the average ICP reading was recorded and compared with previously established control data [44].

### Analysis of the CSF Pathway

Mice (12 months old, *n* = 3 in each group) were anesthetized by an intramuscular injection of a mixture of ketamine (100 mg/kg), xylazine (20 mg/kg), and atropine (2 mg/kg). A small burr hole was drilled into the right parietal bone, 1 mm lateral to the bregma. The head was fixed to a stereotaxic apparatus, and the needle of a microsyringe was inserted into the right lateral ventricle 3 mm from the brain surface through the burr hole. Indian ink (4 µl, 20∼50-nm particle size; Pelikan AG, Hanover, Germany) was slowly injected into the right lateral ventricle at a speed of 60 µl/h. Ten minutes after the injection, the mice were decapitated. The brain was quickly removed, and the dyed meningeal vessels were photographed. The brain was then cut into pieces along the sagittal plane to trace the ink flow within the ventricles.

### Brain Water Content

Brains were removed from the animals (12 months old, *n* = 4 in each group) as described above, weighed for the wet weight, and then dried in an oven at 95°C for 48 h to obtain the dry weight. The water content was calculated by the following equation described elsewhere [45].

Water content (%)  =  [(wet weight-dry weight)/wet weight]×100%

### Blood Pressure

Mice (12 months old, *n* = 3 in each group) were anesthetized by inhalation of 1% isoflurane. The mean blood pressure and heart rate of anesthetized mice were measured using a blood pressure monitor (Model MK-2000; Muromachi Kikai, Tokyo, Japan).

### Serum Biochemical Parameters

Blood samples collected from mice (12 months old, *n* = 3 in each group) using submandibular sticks were used to produce serum, and analyzed by the Taiwan Mouse Clinic (Taipei, Taiwan). Levels of total cholesterol, triglyceride, glucose, magnesium, calcium, creatinine, C-reactive protein, alanine aminotransferase, creatinine kinase, and amylase were determined by the Dri-Chem clinical chemistry analyzing system (FDC 3500; Fuji, Tokyo, Japan) following the manufacturer's protocol. The osmolarity of the serum was determined using the micro-osmometer (Advanced model 3300; Advanced instrument, Norwood, MA, USA).

### Motor Function

Motor coordination was assessed by performance on a rotarod apparatus (UGO BASILE, Viale G. Borghi, Comerio, Italy). From the age of 5 months, mice were first trained on the rotarod device before testing. This training involved slowly increasing the speed of the rotarod device from 4 to 40 rpm over a time course of 10 min. These training sessions were repeated three times prior to testing. As a result of this training, it was determined that a speed of 24 rpm for 2 min was sufficient to distinguish between mice with enlarged ventricles compared to those with normal-sized ventricles [46].

Following the initial training and testing, mice were retested three times in the first week of each month until they reached the age of 24 months when the study was terminated. The time interval taken before the mouse fell from the rotarod was automatically measured within the time interval of 2 min. The maximum retention interval recorded by each mouse was used for comparisons between individuals.

### Gait

The gait of control (*n* = 5) and mutant (*n* = 10) mice aged 20∼24 months was analyzed using a CatWalk gait automatic analyzer (Noldus Information Technology, Leesburg, VA, USA). The position, timing, and dimensions of each footfall were recorded by a video camera positioned underneath a glass plate. The stride distance and position of each paw from the mid-line was recorded and analyzed using ImageJ software (Research Services Branch of the National Institute of Mental Health, Bethesda, MD, USA) [47].

### Magnetic Resonance Imaging (MRI)

The ventricle size of control and mutant mice (aged 1, 3, and 24 months) was assessed using micro-MRI. Mice were anesthetized by inhalation of 5% isoflurane at an oxygenation rate of 1 L/min, and maintained in the magnet with 2%∼3% isoflurane at 1 L/min oxygenation [48]. Three dimensional (3D) ventricle and whole-brain images were evaluated using a Bruker, Pharmascan (7-Tesla) scanner (Bruker Biospin, Ettlingen, Germany) as detailed earlier [46]. Sagittal T2-weight rapid acquisition relaxation enhancement (RARE) images (TR = 2000 ms, TEeff = 88.3 ms, FOV = 3 cm, matrix = 256×128) were acquired and used for the location and length determinations of the brain. T2-weighted 3-D RARE scans were acquired axially (TR = 4000 ms, TEeff = 80 ms, Fov = 2 cm, voxel size = 2.86×10.6 cm, matrix = 256×128×64). The regions of interest within the ventricle were manually delineated into the right lateral ventricle, the left lateral ventricle, the 3^rd^ ventricle, the aqueduct, and the 4^th^ ventricle. The voxels assigned to these individual parts of the ventricles were summed and multiplied by the voxel size to obtain their respective volumes, and the total ventricular volume represents the sum of these separate measurements. All images were processed using a manual tracing tool and edge editing function provided by ANALYZE (Biomedical Imaging Resource, Mayo Foundation, Rochester, MN, USA).

### Urination Test

Control and mutant mice aged 20∼24 months were subjected to mild stress on rotarod at a fixed speed of 4 rpm for 2 min. The amount of voided urine was collected on a piece of Whatman chromatography paper (3 mm thick, 52.5 cm^2^) placed on top of the sensor plate of the rotarod. Each animal was tested three times between 14:00 and 16:00 on three consecutive days at monthly intervals. Urine frequency was calculated by dividing the number of tests in which urination occurred by the total number of tests. The urine-soaked area of each filter paper was scanned, quantified by ImageJ, and transformed into a volume of urine based on a carefully calibrated standard curve (Supplementary Fig. S3).

### Cognitive Function

Mice (20∼24 months old) were subjected to fear conditioning trials associated with an auditory cue followed by an electric shock (Med Associates, Saint Albans, VT, USA). In these trials, a mouse was allowed to explore the conditioning chamber for 2 min. After this period, they were subjected to a 30-s broadcast of white noise (90 dB) followed by an electric shock to the feet for 2 s. For the contextual test, mice were returned to the same training chamber for 5 min without an electric shock. For the cued test, mice were placed in a similar chamber (but lacking the steel rods used to deliver the electric shock) for 3 min, after which they were then exposed to the 90-dB white noise for 3 min. Their response to these test conditions was observed, and the duration of time spent motionless was recorded. Trials were conducted 1 and 24 h after the initial training session to assess short- and long-term memory, respectively [49].

### Brain Tissue Preparation, Immunohistochemical Analyses, and Quantitation

Three to six mice at 3, 6, 18, and 24 months of age from each group were anesthetized by an intraperitoneal injection of sodium pentobarbital (80 mg/kg) prior to intracardial perfusion with 4% paraformaldehyde in 0.1 M phosphate buffer (PB, pH 7.4). Following perfusion, the brains were removed and fixed for a further 24 h in the same solution. After this period, brains were placed in a 30% sucrose solution made up in 0.1 M PB. The tissue was frozen, and 20-µm cryostat sections were taken (HM430, Microm, Germany). Those containing the septum, striatum, and cortex (interaural 4.66 mm/bregma 0.86 mm to interaural 4.18 mm/bregma 0.38 mm) were used for Nissl staining and immunohistochemistry employing the avidin-biotin peroxidase complex (ABC) method [46]. In Nissl-stained brain sections, neurons appeared round and light purple while glia cells appeared dark with condensed nuclei. Astrocytes and microglia were identified by immunohistochemistry using a polyclonal antibody to GFAP (1∶2000, Sigma, St Louis, MO, USA) or Iba1 (1∶1000, Wako Pure Chemical Industries, Osaka, Japan), respectively. Because the Iba1 antibody recognizes all stages of microglia, these cells were classified according to previously defined morphological features [50], [51]: (1) ramified (or resting) microglia have a round cell body with thin cytoplasm and fine processes producing a long/short axis ratio of 1∶1; (2) rod-like (or activated) microglia are hypertrophic with thick cytoplasm and thick short processes producing a long/short axis ratio of <3∶1; and (3) elongated/bipolar (or chronically activated) microglia have an elongated cell body with bipolar thick processes producing a long/short axis ratio of ≥3∶1 (see Supplementary Table S4). Cells were counter-stained with either an anti-NeuN antibody (1∶500; Chemicon, Temecula, CA, USA) as previously described [52] or Hoechst 33342. Optical (Carl Zeiss, Oberkochen, Germany), and laser confocal (LSM510, Carl Zeiss) microscopy was used to quantify at least 6000 cells from each brain section.

### Genetic Analysis

ENU-treated mice originating from a B6 genetic background, were outcrossed to C3H mice for genotyping. DNA from these outbred offspring was extracted using a Genomic DNA Purification Kit (Promega, Madison, WI, USA). Single nucleotide polymorphism (SNP) genotyping was carried out by the National Genotyping Center at Academia Sinica using the SEQUENOM MassARRAY® System (Sequenom, San Diego, CA, USA). Marker information used for gene mapping can be found at Ensembl (http://www.ensembl.org/Mus_musculus/index.html) and is listed in Supplementary Table S5. The recombination frequency was calculated and analyzed using MapManager QTXb06 (Manly et al., 2001). A χ*^2^* square test and the relative risk ratio were used to evaluate the susceptibility of the affected allele.

## Supporting Information

Figure S1 2D-MRI analyses of a wildtype C57BL/6J mouse (A) and a p23-ST1 mouse (B) at 3 months old. The representative p23-ST1 mouse showed enlargement of the lateral ventricles.
(0.75 MB TIF)

Figure S2 p23-ST1 mice which exhibited normal bodyweight (A) and locomotor activity (B).
(0.44 MB TIF)

Figure S3 Standard curve for the measurement of urinary volume on 3-mm Whatman chromatography paper.
(0.43 MB TIF)

Figure S4 Linkage analysis for hydrocephalus by whole-genome screening. The B6 genotype frequency, defined as the percentage of individual animals that contained at least one copy of the B6 allele, of mutant and control mice was analyzed by whole-genome screening using 287 SNP markers.
(0.56 MB TIF)

Figure S5 Genetic map of p23-ST1 mice at the linked locus. Percentages of the B6 allelic frequency at chromosomes 4, 14, and 19 were analyzed. On chromosome 4, the percentages of the B6 allelic frequency in mutant mice (≥ mean +8 SDs, n = 56; open circles) reached 70%; in contrast, in control mice (≤ mean −2 SDs, n = 8; filled circles), it was only 35% in a linked region (flanked by rs13477838 and rs13477993) on chromosome 4.
(0.49 MB TIF)

Figure S6 Fine mapping of the genetic map at the linked locus of chromosome 4. Percentages of B6 allele frequency (A) and B6 homozygosity (B) in the linked locus (rs13477838∼rs13477993) on chromosome 4 in mutant and control mice were analyzed using additional SNP (rs13477964, rs6224690, rs27565906, rs134779969, rs6228499, and rs6334695) and STRP (D4Mit249, D4Mit16, D4Mit204, and D4Mit71) markers. Percentages of both B6 allele frequency and B6 homozygosity in the linked region increased with the phenotype of enlargement of the ventricles (from 60% (ventricle size, mean +5 SDs) to 70% (mean +8 SDs)), while only a low percentage (30%) was detected in control mice.
(0.52 MB TIF)

Table S1 3D-MRI analysis of wildtype C57BL/6J and C3H/HeJ mice at the age of 3 months
(0.26 MB TIF)

Table S2 Serum biochemical parameters and osmolarity of control and mutant mice. Blood samples were collected from mice at the age of 12 months (n = 3 in each group) to produce serum and analyzed for the levels of total-cholesterol (TCHO); triglyceride (TG), glucose (GLU), magnesium (Mg), calcium (Ca), creatinine (CRE), C-reactive protein (CRP), alanine aminotransferase (ALT), creatinine kinase (CPK), amylase (AMYL) and osmolarity. Data are presented as the mean±SEM in each group.
(0.25 MB TIF)

Table S3 A Chi-square test to assess the linkage of the B6 allele at rs3091114 on chromosome 4 to hydrocephalus. The two-by-two table demonstrates that the mutant B6 allele in this region was significantly associated with the disease phenotype with χ^2^ = 16.141, *p*<0.001.
(0.23 MB TIF)

Table S4 Morphological characteristics of Iba1-positive microglia. Iba1-positive microglia were classified according to their morphological features: (A) Ramified microglia (resting); (B) rod-like/amoeboid microglia (activated); (C) elongated/bipolar microglia (chronically activated). Representative images of the schematic illustrations (1), immunohistochemistry images (2), and 3D reconstructed images of immunofluorescence staining (3) are presented.
(1.09 MB TIF)

Table S5 Primers used for gene mapping on chromosome 4
(0.69 MB TIF)
