# Supplementary figures and images for: Longitudinal Evaluation of an N-Ethyl-N-Nitrosourea-Created Murine Model with Normal Pressure Hydrocephalus
Source: PLoS One. 2009 Nov 17;4(11):e7868. doi: 10.1371/journal.pone.0007868 (PMC2774278; doi:10.1371/journal.pone.0007868)

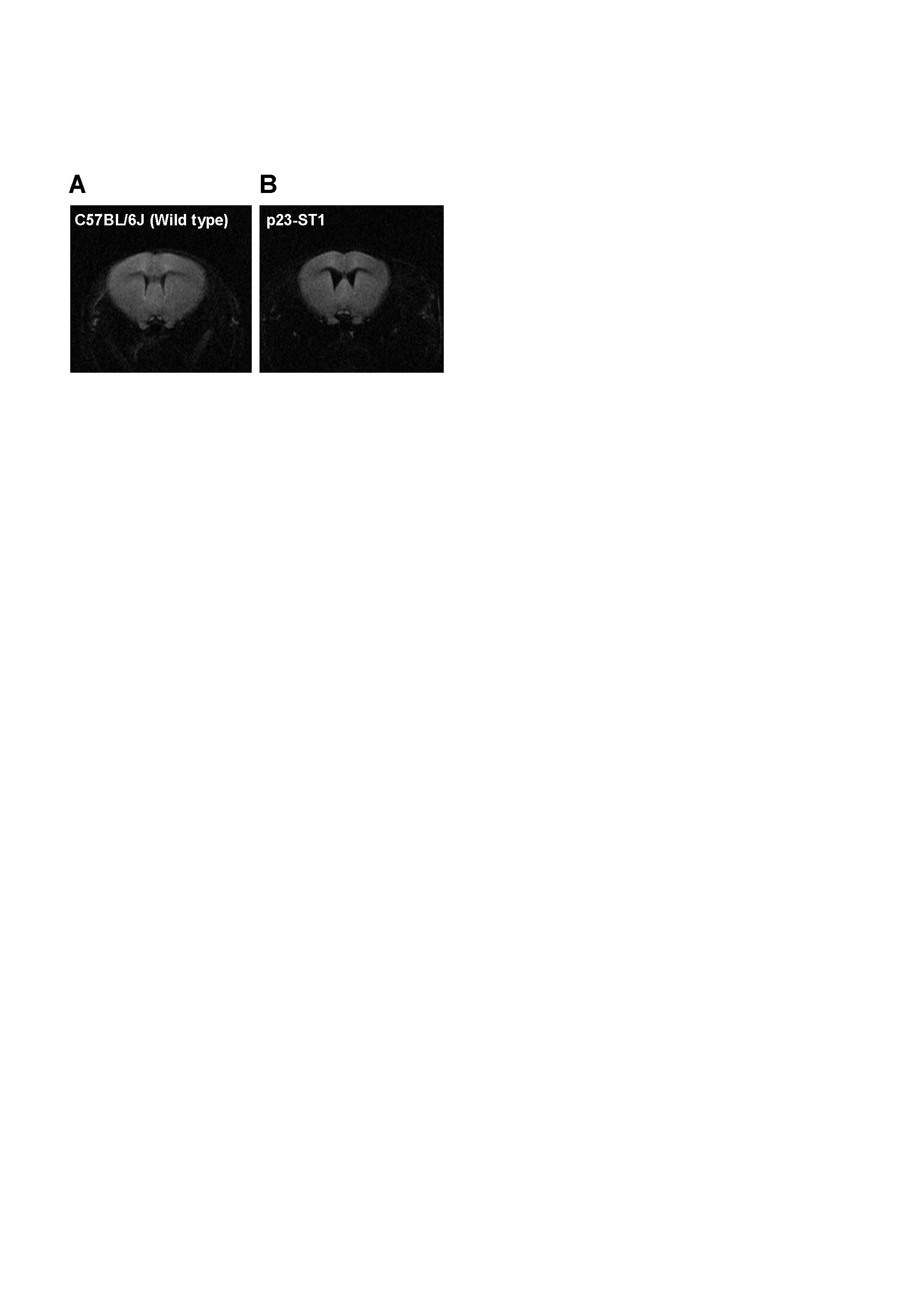

Supplement: Figure S1 — 2D-MRI analyses of a wildtype C57BL/6J mouse (A) and a p23-ST1 mouse (B) at 3 months old. The representative p23-ST1 mouse showed enlargement of the lateral ventricles. (0.75 MB TIF) [file pone.0007868.s001.tif]

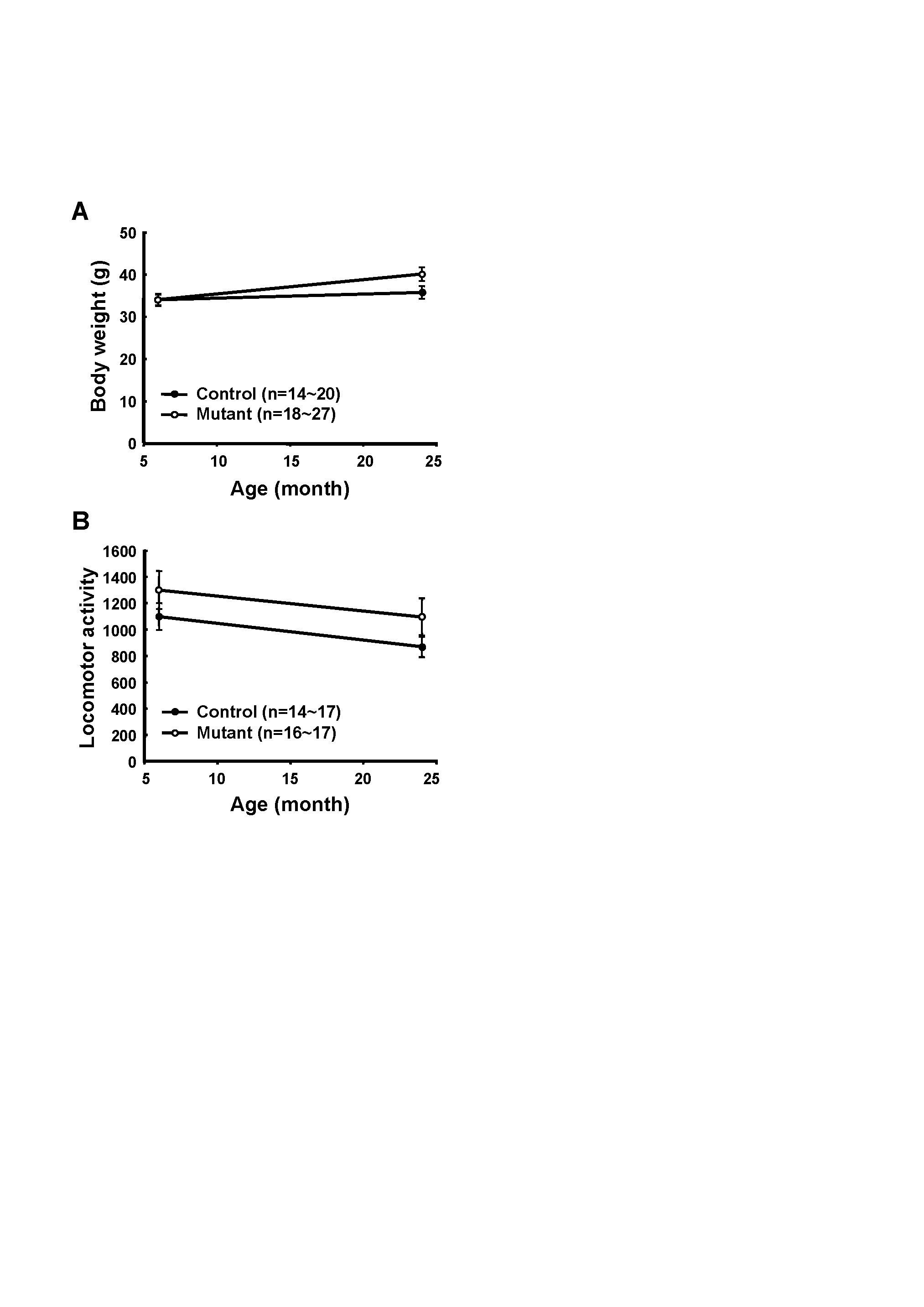

Supplement: Figure S2 — p23-ST1 mice which exhibited normal bodyweight (A) and locomotor activity (B). (0.44 MB TIF) [file pone.0007868.s002.tif]

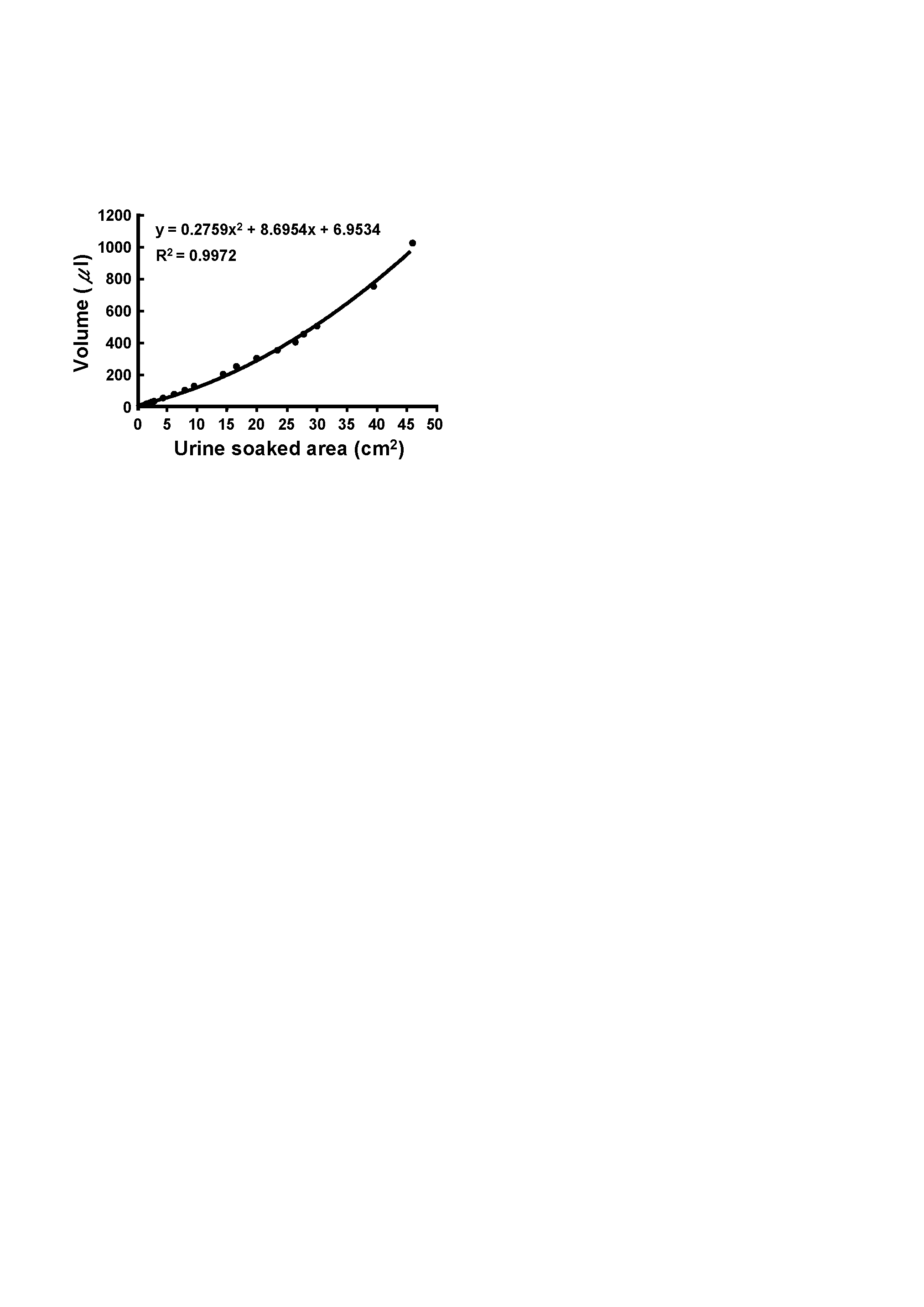

Supplement: Figure S3 — Standard curve for the measurement of urinary volume on 3-mm Whatman chromatography paper. (0.43 MB TIF) [file pone.0007868.s003.tif]

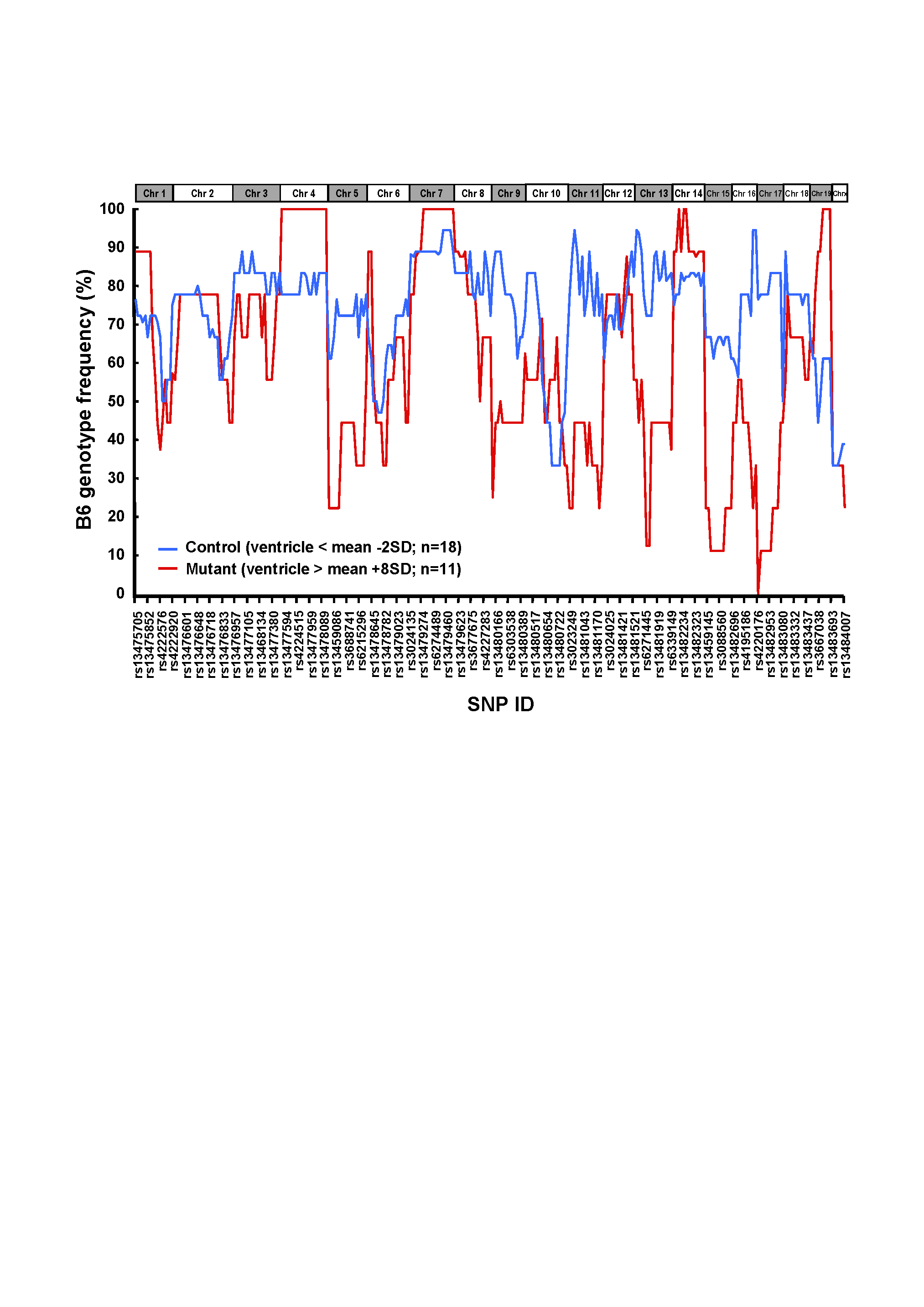

Supplement: Figure S4 — Linkage analysis for hydrocephalus by whole-genome screening. The B6 genotype frequency, defined as the percentage of individual animals that contained at least one copy of the B6 allele, of mutant and control mice was analyzed by whole-genome screening using 287 SNP markers. (0.56 MB TIF) [file pone.0007868.s004.tif]

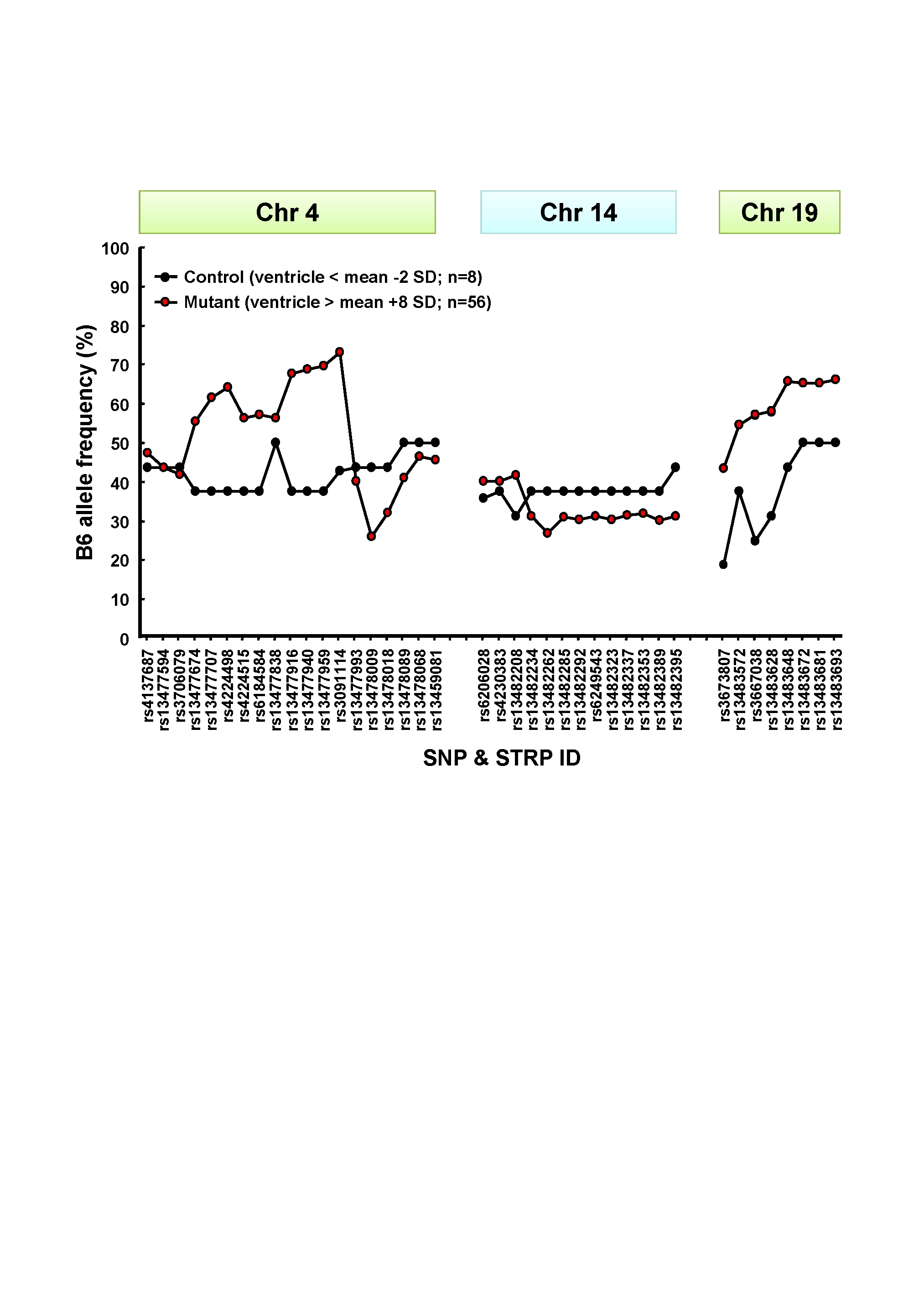

Supplement: Figure S5 — Genetic map of p23-ST1 mice at the linked locus. Percentages of the B6 allelic frequency at chromosomes 4, 14, and 19 were analyzed. On chromosome 4, the percentages of the B6 allelic frequency in mutant mice (≥ mean +8 SDs, n = 56; open circles) reached 70%; in contrast, in control mice (≤ mean −2 SDs, n = 8; filled circles), it was only 35% in a linked region (flanked by rs13477838 and rs13477993) on chromosome 4. (0.49 MB TIF) [file pone.0007868.s005.tif]

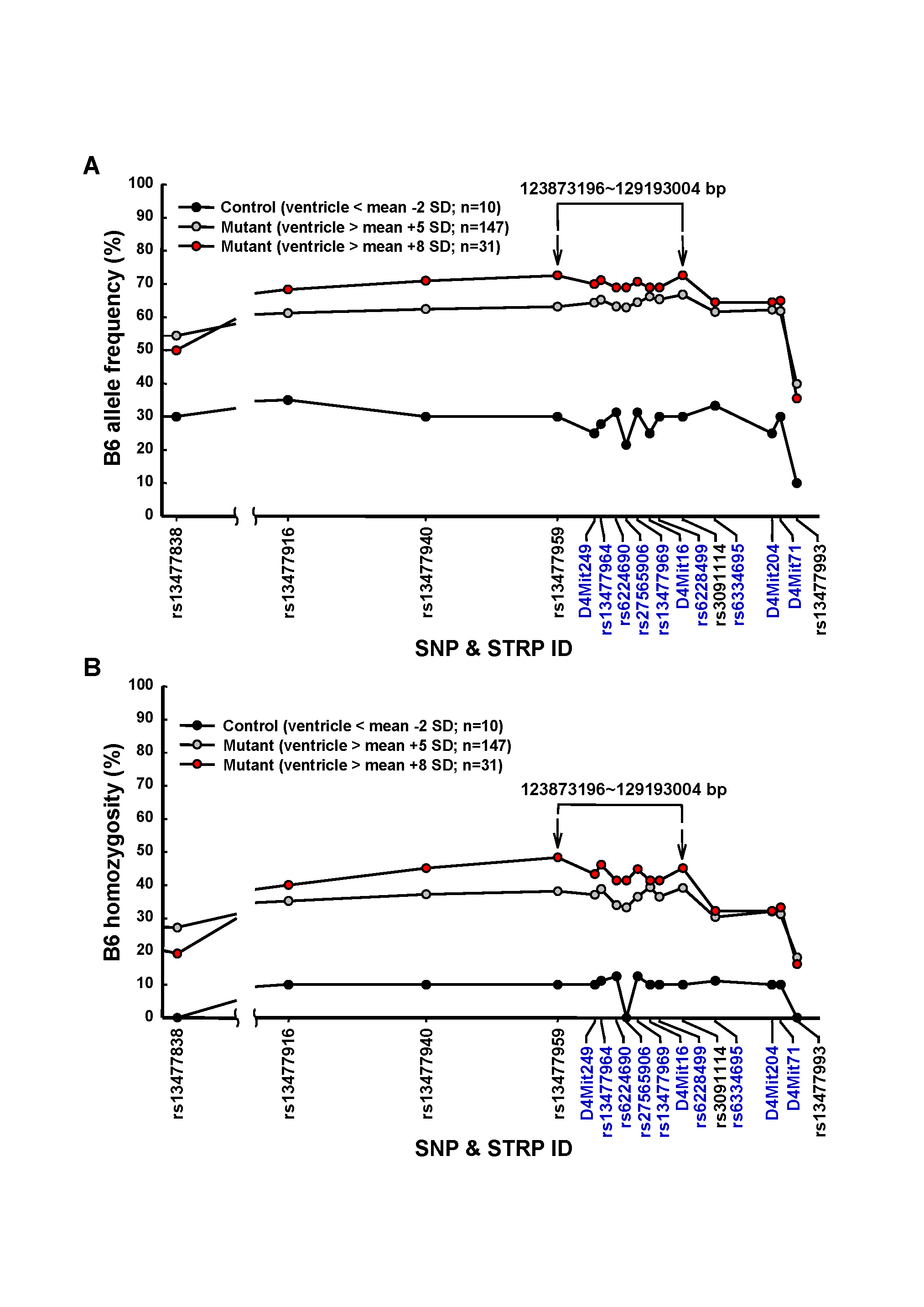

Supplement: Figure S6 — Fine mapping of the genetic map at the linked locus of chromosome 4. Percentages of B6 allele frequency (A) and B6 homozygosity (B) in the linked locus (rs13477838∼rs13477993) on chromosome 4 in mutant and control mice were analyzed using additional SNP (rs13477964, rs6224690, rs27565906, rs134779969, rs6228499, and rs6334695) and STRP (D4Mit249, D4Mit16, D4Mit204, and D4Mit71) markers. Percentages of both B6 allele frequency and B6 homozygosity in the linked region increased with the phenotype of enlargement of the ventricles (from 60% (ventricle size, mean +5 SDs) to 70% (mean +8 SDs)), while only a low percentage (30%) was detected in control mice. (0.52 MB TIF) [file pone.0007868.s006.tif]

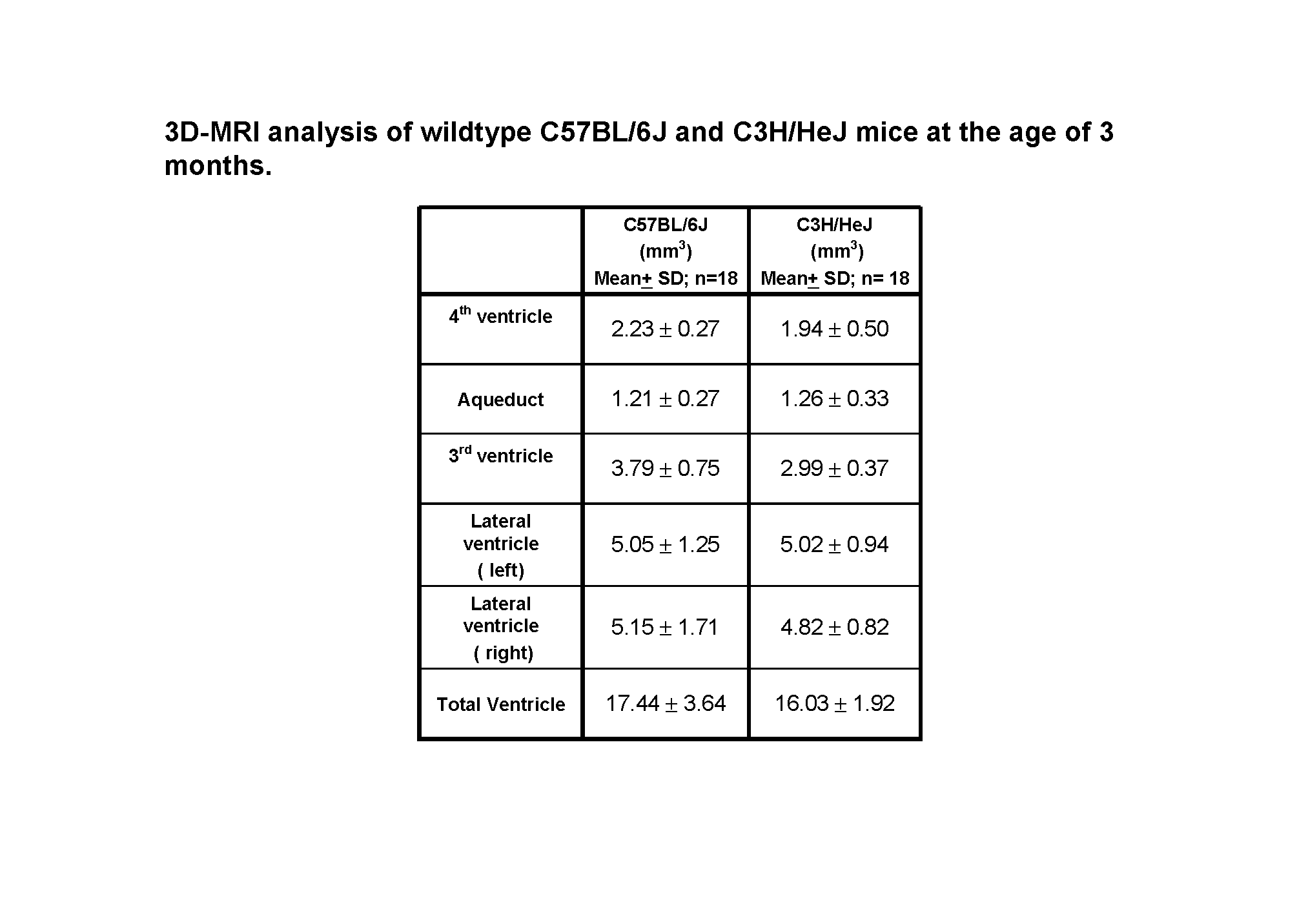

Supplement: Table S1 — 3D-MRI analysis of wildtype C57BL/6J and C3H/HeJ mice at the age of 3 months (0.26 MB TIF) [file pone.0007868.s007.tif]

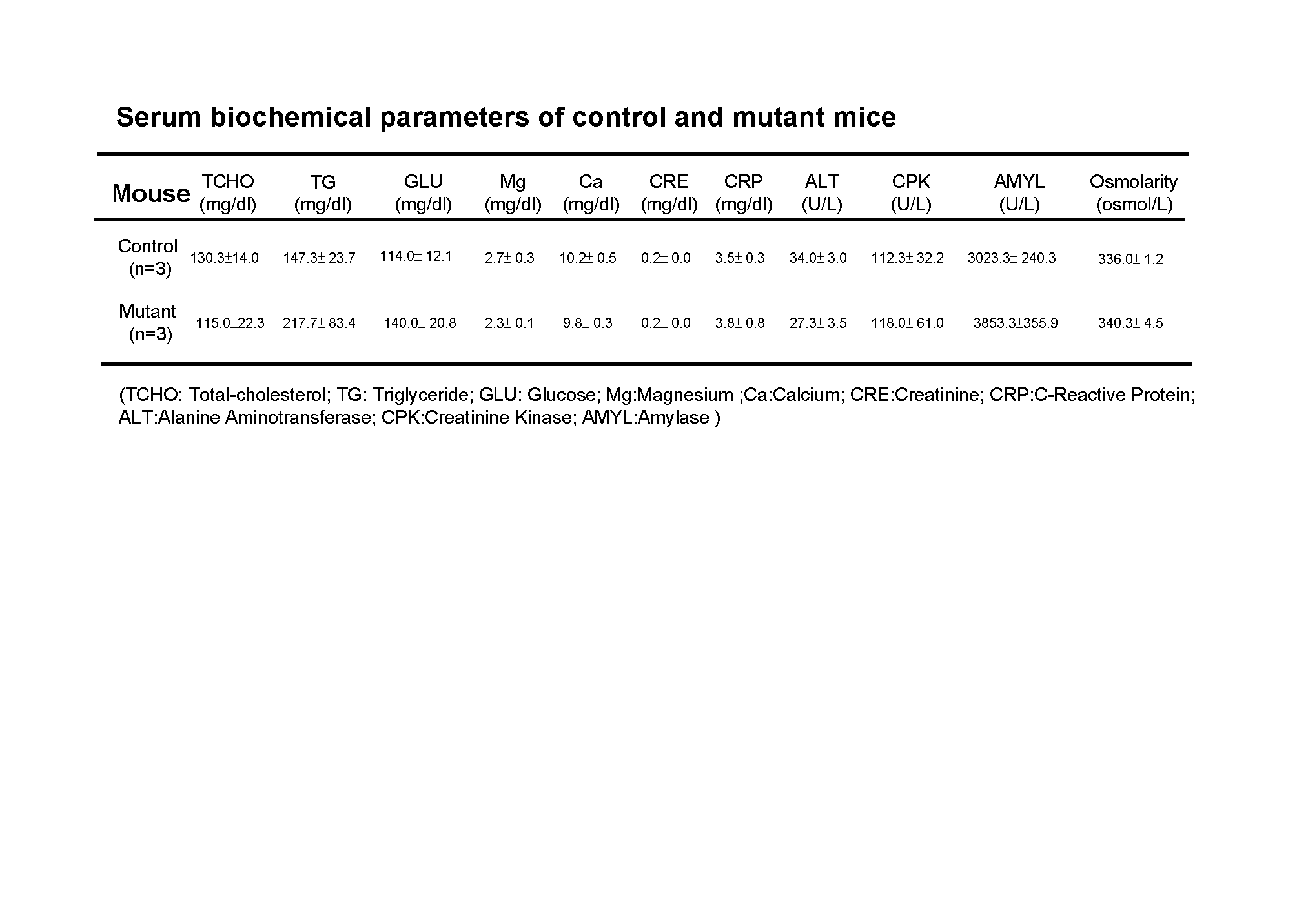

Supplement: Table S2 — Serum biochemical parameters and osmolarity of control and mutant mice. Blood samples were collected from mice at the age of 12 months (n = 3 in each group) to produce serum and analyzed for the levels of total-cholesterol (TCHO); triglyceride (TG), glucose (GLU), magnesium (Mg), calcium (Ca), creatinine (CRE), C-reactive protein (CRP), alanine aminotransferase (ALT), creatinine kinase (CPK), amylase (AMYL) and osmolarity. Data are presented as the mean±SEM in each group. (0.25 MB TIF) [file pone.0007868.s008.tif]

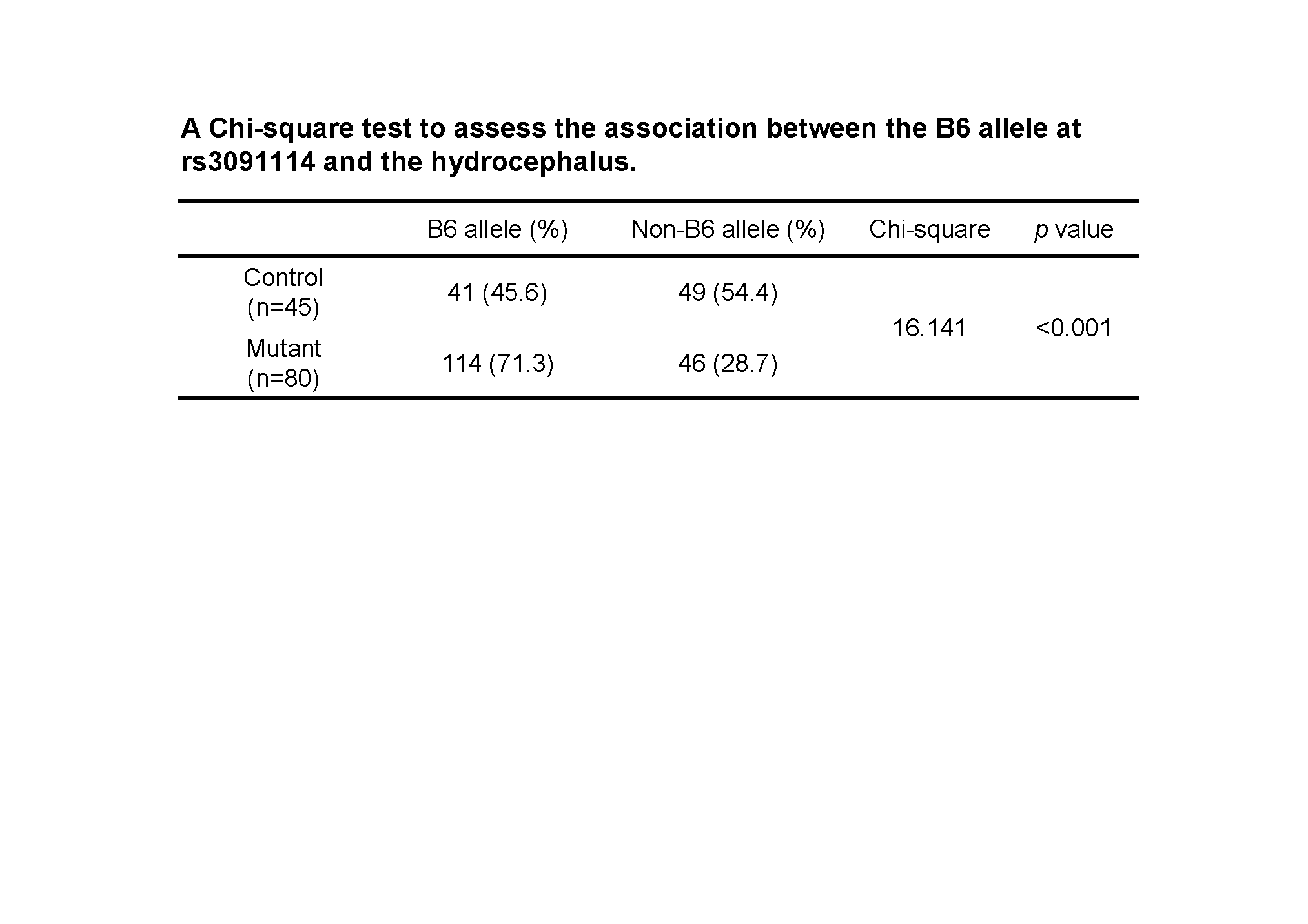

Supplement: Table S3 — A Chi-square test to assess the linkage of the B6 allele at rs3091114 on chromosome 4 to hydrocephalus. The two-by-two table demonstrates that the mutant B6 allele in this region was significantly associated with the disease phenotype with χ2 = 16.141, p<0.001. (0.23 MB TIF) [file pone.0007868.s009.tif]

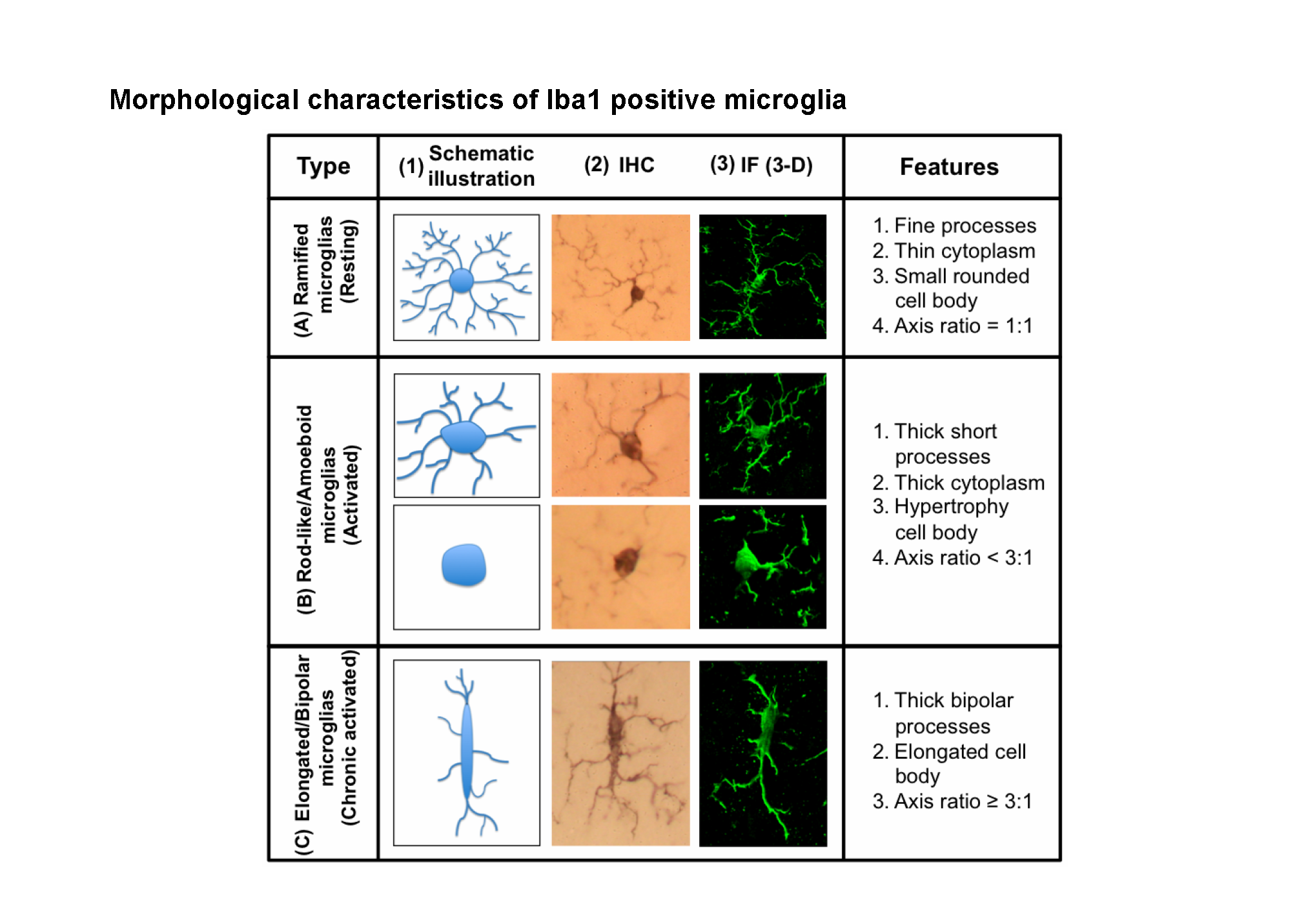

Supplement: Table S4 — Morphological characteristics of Iba1-positive microglia. Iba1-positive microglia were classified according to their morphological features: (A) Ramified microglia (resting); (B) rod-like/amoeboid microglia (activated); (C) elongated/bipolar microglia (chronically activated). Representative images of the schematic illustrations (1), immunohistochemistry images (2), and 3D reconstructed images of immunofluorescence staining (3) are presented. (1.09 MB TIF) [file pone.0007868.s010.tif]

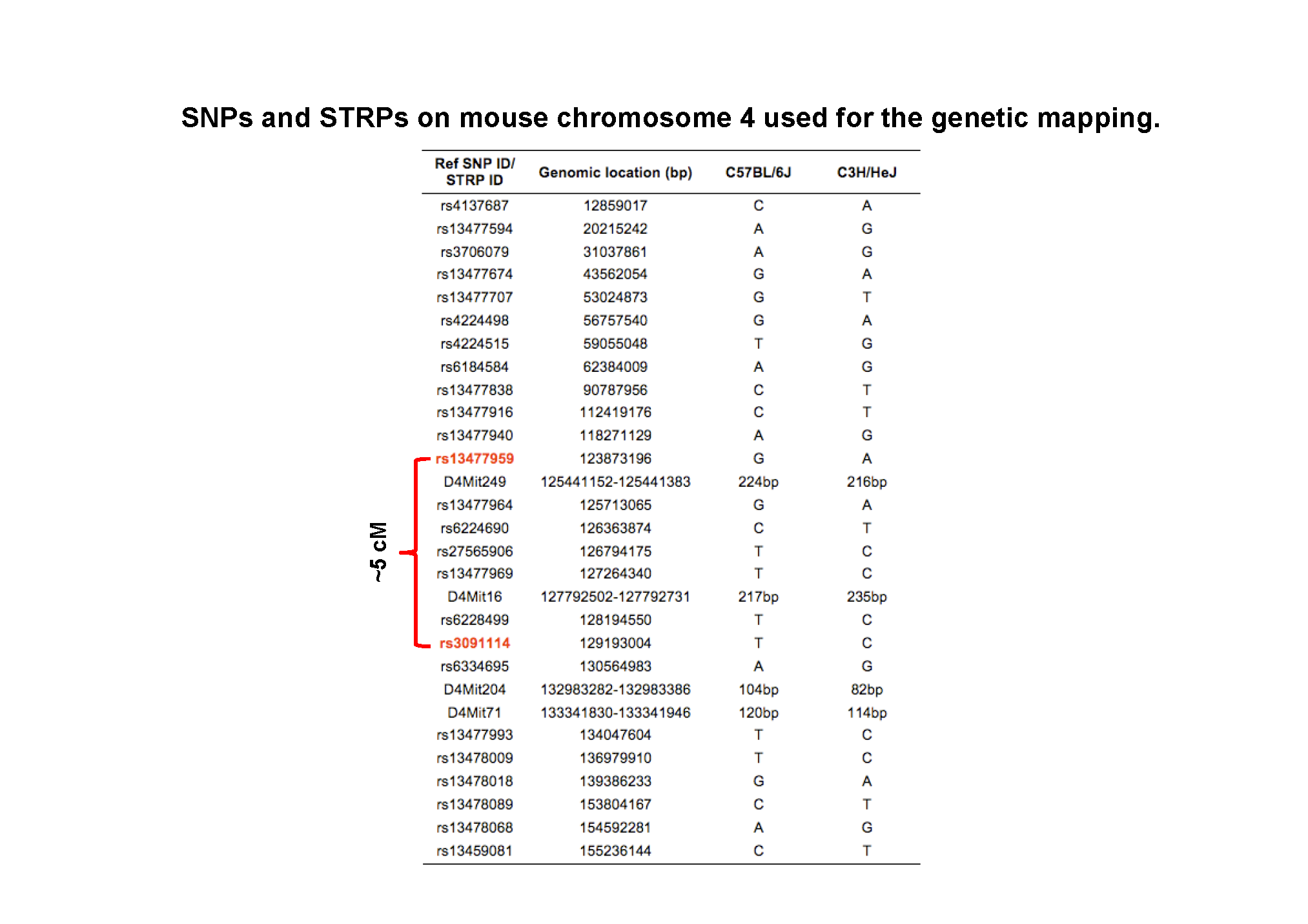

Supplement: Table S5 — Primers used for gene mapping on chromosome 4 (0.69 MB TIF) [file pone.0007868.s011.tif]
